# Supplementary material for: Length-dependent poleward flux of sister kinetochore fibers promotes chromosome alignment
Source: Cell Rep. 2022 Aug 3;40(5):111169. doi: 10.1016/j.celrep.2022.111169 (PMC9364240; doi:10.1016/j.celrep.2022.111169)
Supplement: Document S1. Figures S1–S7 [file mmc1.pdf]

Cell Reports, Volume *40*

Supplemental information

Length-dependent poleward flux of sister kinetochore fibers promotes chromosome alignment

Patrik Risteski, Domagoj Božan, Mihaela Jagrić, Agneza Bosilj, Nenad Pavin, and Iva M. Tolić

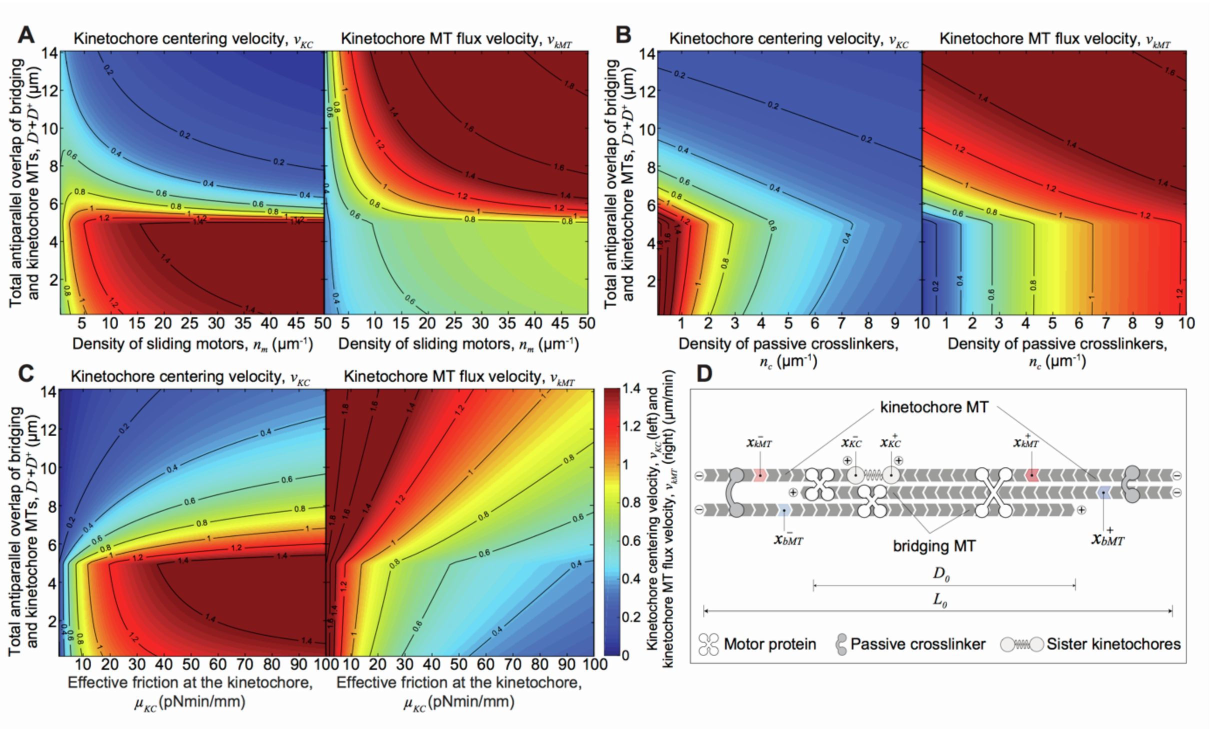

Figure S1. Centering velocity and k-fiber flux for different parameters, Related to Figure 1. Kinetochore centering velocity (left) and kMT flux velocity (right) for different values of motor density (**A**), crosslinker density (**B**), and effective friction at the kinetochore (**C**), and different values of the length of total antiparallel overlap of bMTs and kMTs. In all cases, faster centering velocities correlate with slower k-fiber flux velocities and vice versa and thus we comment transitions for centering velocities only. (**A**) Data shows two distinct regimes of centering velocity: fast centering velocities for shorter overlaps (red region) which sharply decreases when the overlap length exceeds 4 μm (blue region). This abrupt change occurs when the shorter k-fiber loses connection with antiparallel bMT and thus there is no motor force that opposes centering movement. Transition between these two regimes is less abrupt for lower values of motor densities ($n_\mathrm{m} < 10\ \mu\mathrm{m}^{-1}$). (**B**) Similar to panel **A**, fast centering velocities are obtained for shorter overlaps (red region). However, the centering velocity decreases with the increase in crosslinker density, irrespective of the overlap length (blue region). (**C**) Fast centering velocities are obtained for shorter overlaps (red region) and the centering velocity increases with the increase in effective friction. For low values of effective friction, both the centering velocity and the k-fiber flux velocity retain similar values regardless of the overlap length. Bar on the right denotes the relationship between color and velocity values for panels **A**-**C**. (**D**) Top: Scheme of the model. KMTs extend from the edges toward elastically connected kinetochores and bMTs extend from the edges towards each other. Motor proteins connect antiparallel MTs, while passive crosslinkers connect parallel MTs. Total lengths of antiparallel

and parallel MT overlaps are denoted as D_0 and L_0, respectively. Positions of sister kinetochores are marked on the x-axis. Positions of k-fibers (red) and bridging fibers (blue) are taken as arbitrary positions along their lattice and are also marked on the x-axis. Superscripts + and – denote the right and left sides, respectively. Bottom: Legend describing symbols for different elements of the spindle in the scheme. Parameters for all panels are given in **Figure 1B** and the initial position of kinetochores is $x_{\mathrm{KC}} = -2$ μm, if not stated otherwise.

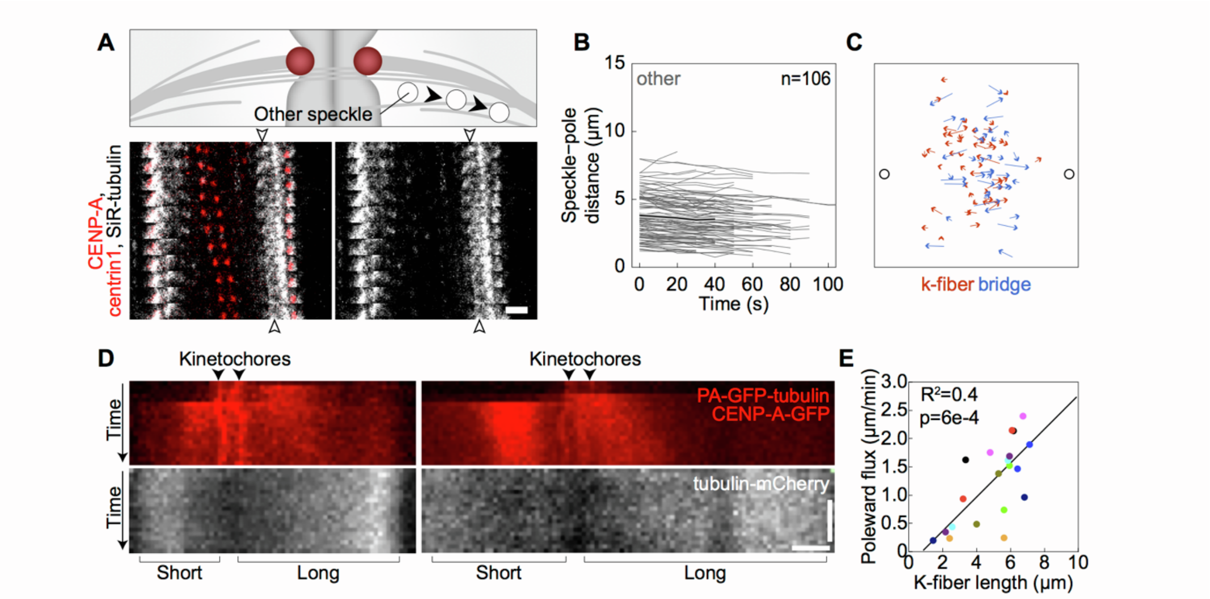

Figure S2. K-fiber poleward flux correlates with k-fiber length, Related to Figure 2. (**A**) Scheme of "other" speckles for which it could not be determined the type of MT they belong to (top). Montage over time demonstrating the movement of this group of speckles. Merge (left); tubulin channel only (right). Arrowheads mark starting and ending positions of the tracked speckle. Scale bar: 2 μm. (**B**) Distance of "other" speckles from the pole over time in untreated cells. Gray lines show individual speckles. Black line; mean. Gray area; SEM (**C**) Examples of trajectories of speckles belonging to k-fibers (red) and bridging fibers (blue) within 30 s of their movement. Arrows are pointing towards corresponding direction. Black circles; spindle poles. (**D**) Kymographs retrieved by pole-to-pole segmented lines in U2OS cells stably co-expressing PA-GFP-α-tubulin (red), CENP-A-GFP (red) and mCherry-α-tubulin (gray) during poleward motion of the photoactivated spots on shorter and longer sister k-fibers. Horizontal scale bar, 2 μm; vertical scale bar, 20 s. (**E**) Graph shows poleward flux of photoactivated spots in U2OS cells with respect to their corresponding k-fiber length, color-coded for each sister k-fiber pair as in **Figure 2I**.

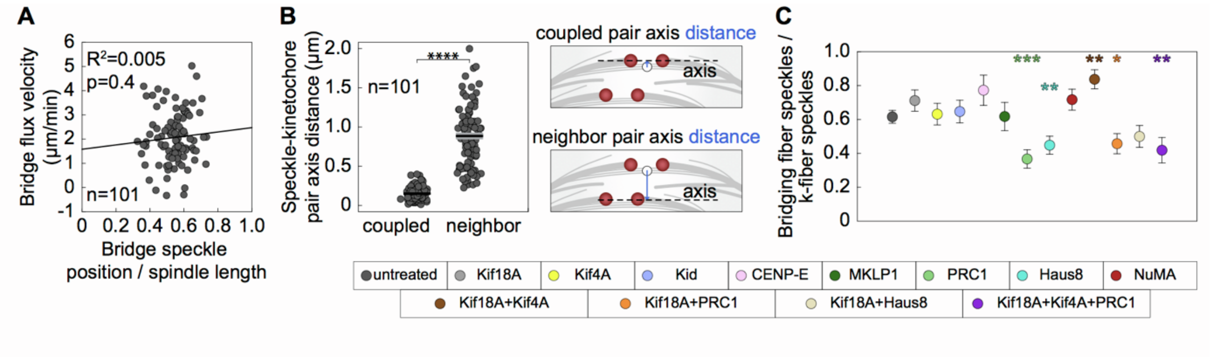

Figure S3. Characterization of the bridging fiber poleward flux, Related to Figure 3. (**A**) Poleward velocity of bridging fiber speckles depending on their relative starting speckle-pole distance. Starting position of all bridging fiber speckles was close to the position of associated kinetochore pair. (**B**) Distances between tracked bridging fiber speckle and kinetochore pair they were associated to and between tracked bridging fiber speckle and their closest neighboring kinetochore pair. Schematics represent how these distances were measured. Black lines, mean; gray areas, SEM. (**C**) Ratio of tracked speckles within bridging fibers and k-fibers (top) color-coded for corresponding treatments as in legend (bottom). In **C**, each treatment is compared with untreated cells. Treatments include at least three independent experiments. Statistical analysis conducted using t-test (**B**) and two-proportions z-test (**C**); p values as in **Figure 3**.

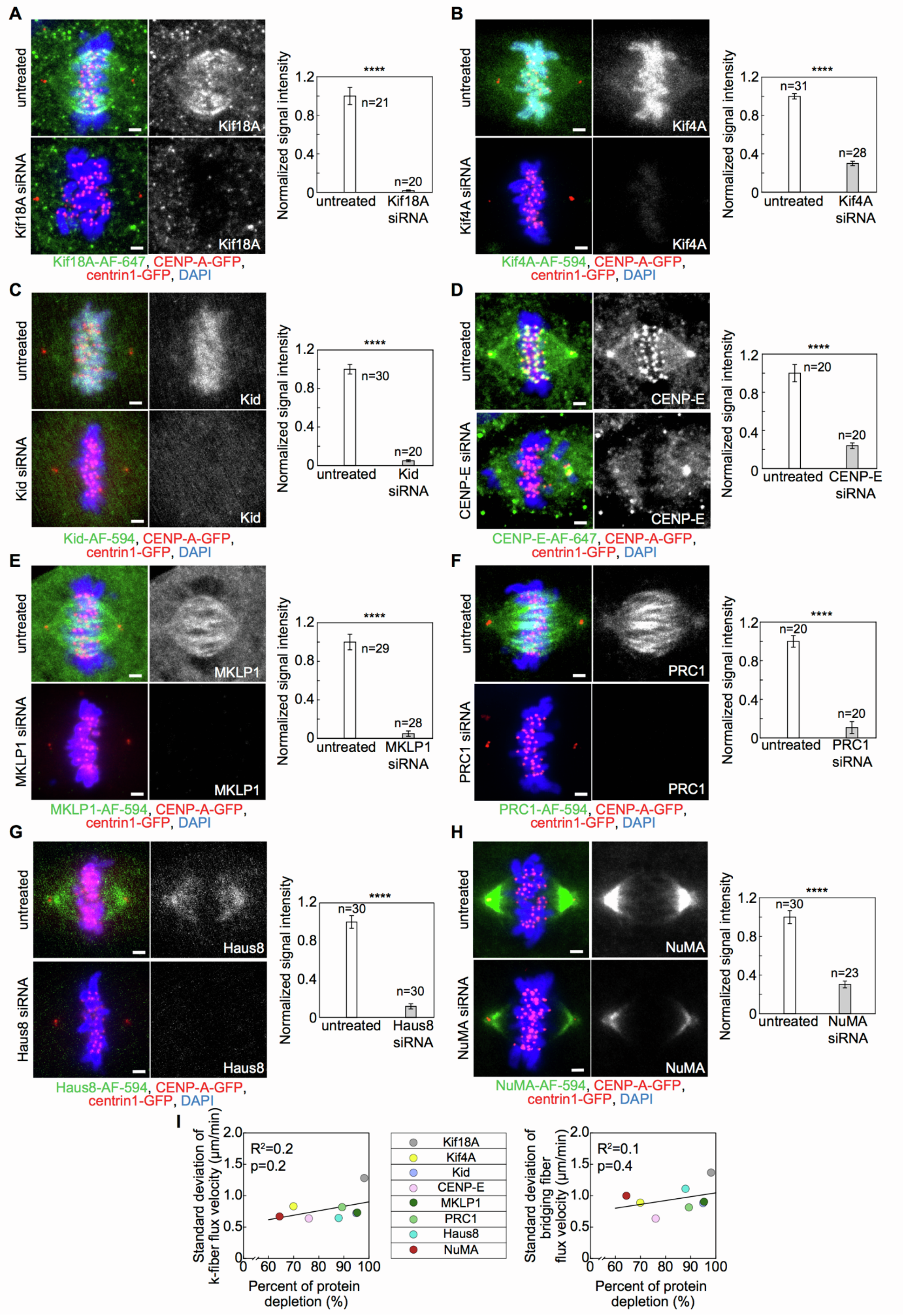

A
untreated
Kif18A siRNA
Kif18A
Kif18A
Normalized signal intensity
****
n=21
n=20
untreated
Kif18A siRNA
Kif18A-AF-647, CENP-A-GFP, centrin1-GFP, DAPI
B
untreated
Kif4A siRNA
Kif4A
Kif4A
n=31
n=28
untreated
Kif4A siRNA
Kif4A-AF-594, CENP-A-GFP, centrin1-GFP, DAPI
C
untreated
Kid siRNA
Kid
Kid
n=30
n=20
untreated
Kid siRNA
Kid-AF-594, CENP-A-GFP, centrin1-GFP, DAPI
D
untreated
CENP-E siRNA
CENP-E
CENP-E
n=20
n=20
untreated
CENP-E siRNA
CENP-E-AF-647, CENP-A-GFP, centrin1-GFP, DAPI
E
untreated
MKLP1 siRNA
MKLP1
MKLP1
n=29
n=28
untreated
MKLP1 siRNA
MKLP1-AF-594, CENP-A-GFP, centrin1-GFP, DAPI
F
untreated
PRC1 siRNA
PRC1
PRC1
n=20
n=20
untreated
PRC1 siRNA
PRC1-AF-594, CENP-A-GFP, centrin1-GFP, DAPI
G
untreated
Haus8 siRNA
Haus8
Haus8
n=30
n=30
untreated
Haus8 siRNA
Haus8-AF-594, CENP-A-GFP, centrin1-GFP, DAPI
H
untreated
NuMA siRNA
NuMA
NuMA
n=30
n=23
untreated
NuMA siRNA
NuMA-AF-594, CENP-A-GFP, centrin1-GFP, DAPI
I
Standard deviation of k-fiber flux velocity (µm/min)
R²=0.2
p=0.2
Percent of protein depletion (%)
Kif18A
Kif4A
Kid
CENP-E
MKLP1
PRC1
Haus8
NuMA
Standard deviation of bridging fiber flux velocity (µm/min)
R²=0.1
p=0.4
Percent of protein depletion (%)

Figure S4. Depletion efficiencies for siRNA treatments, Related to Figure 3. (**A**)-(**H**) Fixed spindles in RPE1 cell line stably expressing CENP-A-GFP and centrin1-GFP (red) in cells immunostained for (**A**) Kif18A (AF-647, green), (**B**) Kif4A (AF-594, green), (**C**) Kid (AF-594, green), (**D**) CENP-E (AF-647, green), (**E**) MKLP1 (AF-594, green), (**F**) PRC1 (AF-594, green), (**G**) Haus8 (AF-594, green) and (**H**) NuMA (AF-594, green) in untreated (upper rows) and corresponding siRNA-treated cells (bottom rows), with DNA stained with DAPI (blue). Left: merge; right: protein of interest (gray). Graphs showing intensities of indicated proteins in untreated (white bars) and siRNA treated (gray bars) cells are given on the right. All values are normalized to the mean intensity value of untreated cells for each protein. All treatments include at least two independent experiments. n; number of cells. Scale bars; 2 μm. All images are maximum intensity projections of five z-planes smoothed with 0.5-pixel-sigma Gaussian blur. (**I**) Standard deviation of k-fiber (left) and bridging fiber (right) flux velocity with respect to the level of protein depletion in corresponding siRNA-treatments (legend). Statistical analysis conducted using t-test; p values as in **Figure 3**.

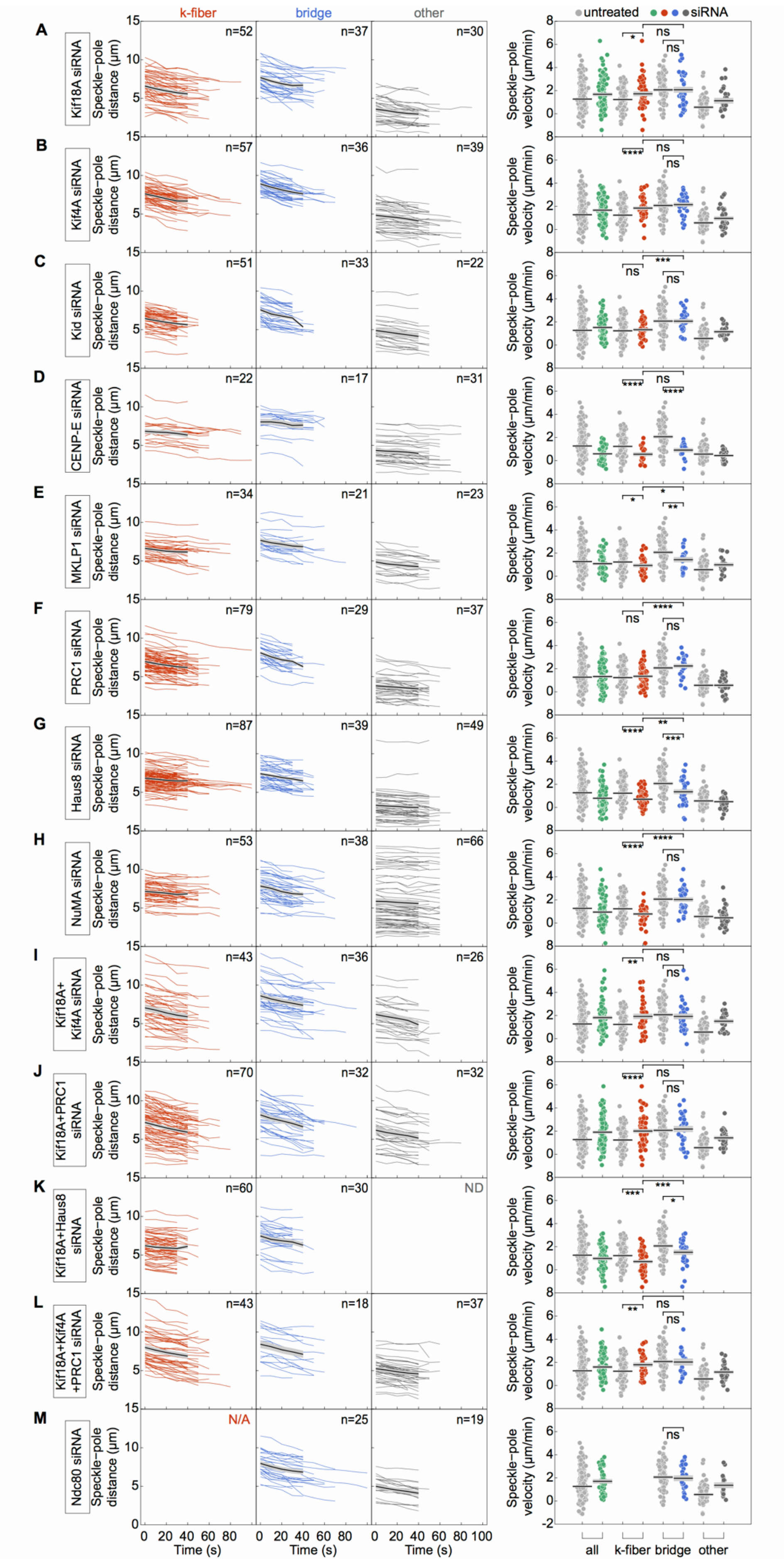
k-fiber
bridge
other
untreated
siRNA
A
Kif18A siRNA
n=52
n=37
n=30
B
Kif4A siRNA
n=57
n=36
n=39
C
Kid siRNA
n=51
n=33
n=22
D
CENP-E siRNA
n=22
n=17
n=31
E
MKLP1 siRNA
n=34
n=21
n=23
F
PRC1 siRNA
n=79
n=29
n=37
G
Haus8 siRNA
n=87
n=39
n=49
H
NuMA siRNA
n=53
n=38
n=66
I
Kif18A+ Kif4A siRNA
n=43
n=36
n=26
J
Kif18A+PRC1 siRNA
n=70
n=32
n=32
K
Kif18A+Haus8 siRNA
n=60
n=30
ND
L
Kif18A+Kif4A +PRC1 siRNA
n=43
n=18
n=37
M
Ndc80 siRNA
N/A
n=25
n=19
Speckle–pole distance (µm)
Speckle–pole velocity (µm/min)
Time (s)
all
k-fiber
bridge
other
ns
*
**
***
****

Figure S5. Comparison of microtubule poleward flux rates per treatment, Related to Figure 3. Poleward flux after depletion of (**A**) Kif18A, (**B**) Kif4A, (**C**) Kid, (**D**) CENP-E (**E**) MKLP1, (**F**) PRC1, (**G**) Haus8, (**H**) NuMA, (**I**) Kif18A+Kif4A, (**J**) Kif18A+PRC1, (**K**) Kif18A+Haus8, (**L**) Kif18A+Kif4A+PRC1 and (**M**) Ndc80. Graphs from left to right show: speckles on kMTs, speckles on bMTs, and other speckles. Colored lines show individual speckles. Black lines; mean. Gray areas; SEM. Poleward velocity of the speckles is shown at the right. Black lines; mean. Gray areas; SEM. One outlier in untreated cells is not shown. All treatments include at least three independent experiments. n; number of measurements. Statistical analysis conducted using t-test; p values as in **Figure 3**.

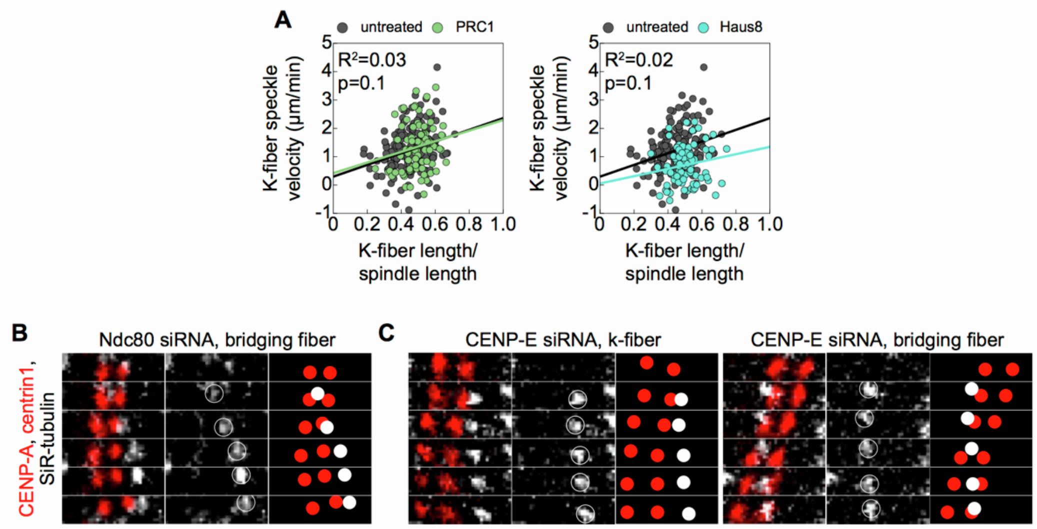

Figure S6. Effect of the bridging microtubule sliding on k-fiber poleward flux, Related to Figure 3. (**A**) Poleward velocity of k-fiber speckles depending on their relative starting speckle-pole distance in PRC1 (left) and Haus8 (right) siRNA-treated cells. (**B**) Montage over time demonstrating the movement of a speckle belonging to the bridging fiber in Ndc80 siRNA treatment. (**C**) Montage over time demonstrating the movement of a speckle belonging to the k-fiber (left) and bridging fiber (right) in CENP-E siRNA treatment. Legend as in **Figure 2B**.

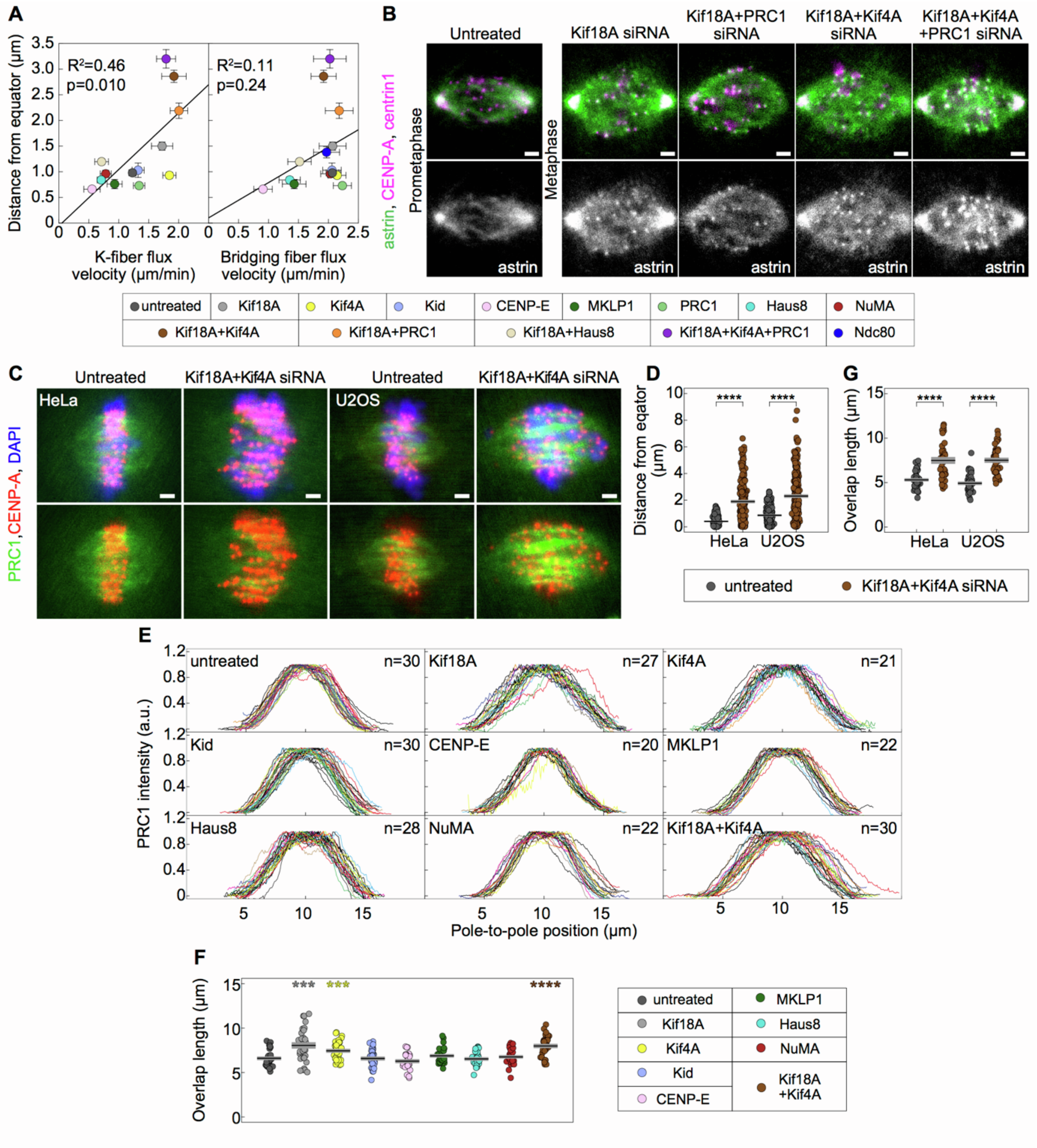

Figure S7. Longer antiparallel overlaps lead to increased k-fiber flux velocity and kinetochore misalignment, Related to Figures 4 and 5. (**A**) Kinetochore distance from equator versus k-fiber (left) and bridging fiber (right) flux velocity in untreated and siRNA-treated cells. (**B**) Fixed spindles in RPE1 cells stably expressing CENP-A-GFP and centrin1-GFP (magenta), immunostained for astrin (AF-594, green) in untreated and treated with Kif18A, Kif18A and PRC1, Kif18A and Kif4A, and Kif18A, Kif4A and PRC1 siRNA (left to right). Top: merge; bottom: astrin (gray). Images are sum intensity projections of five z-planes. (**C**) Fixed spindles in HeLa and U2OS cells stably expressing CENP-A-GFP (red) in untreated

(left) and Kif18A and Kif4A siRNA treated cells (right), immunostained for PRC1 (AF-594, green) and stained with DAPI (blue). Top: merge; bottom: only CENP-A and PRC1. Images are maximum intensity projections of five z-planes. (**D**) Kinetochore distance from equator in untreated and Kif18A and Kif4A siRNA treated HeLa (n = 172 and n = 235 kinetochore pairs) and U2OS (n = 216 and n = 281 kinetochore pairs) cells. Black lines, mean. Gray areas; SEM. (**E**) Normalized pole-to-pole PRC1 intensity profiles of complete spindles for given treatments. Lines correspond to individual spindles. (**F**) Length of individual PRC1-labeled overlaps. siRNA treatments are color-coded according to the legend. Black lines, mean. Gray areas; SEM. (**G**) Length of individual PRC1-labeled overlaps in untreated and Kif18A and Kif4A siRNA treated HeLa (n = 46 and n = 49 PRC1 bundles) and U2OS (n = 47 and n = 41 PRC1 bundles) cells. Black lines, mean. Gray areas; SEM. In **F**, each treatment is compared with untreated cells. Treatments in **A**, **D**, **F**, **G** are color-coded according to the legend. Statistical analysis conducted using t-test in **F**, **G**, and the Mann-Whitney test in **D**; p values as in **Figure 3**.
