# Supplementary material for: Length-dependent poleward flux of sister kinetochore fibers promotes chromosome alignment
Source: Cell Rep. 2022 Aug 3;40(5):111169. doi: 10.1016/j.celrep.2022.111169 (PMC9364240; doi:10.1016/j.celrep.2022.111169)
Supplement: Document S2. Article plus supplemental information [file mmc7.pdf]

Cell Reports

Article

Length-dependent poleward flux of sister kinetochore fibers promotes chromosome alignment

Graphical abstract

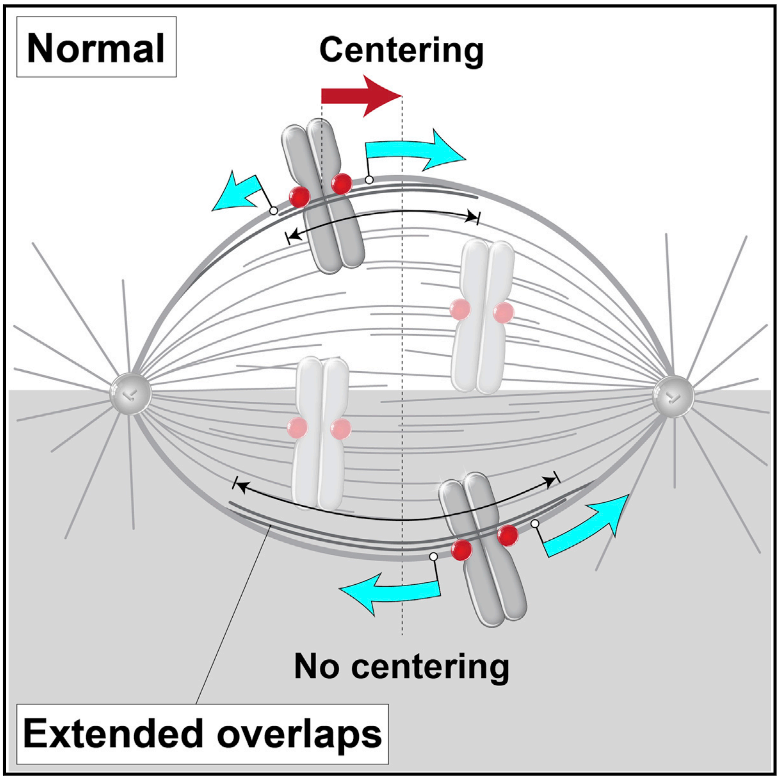

Highlights

- A flux-driven centering model explains chromosome alignment at the spindle equator
- Kinetochore fiber poleward flux is driven by overlap microtubules
- Kinetochore centering requires faster flux of the longer sister kinetochore fiber
- Chromosome alignment depends on the overlap length of bridging microtubules

Authors

Patrik Risteski, Domagoj Božan, Mihaela Jagrić, Agneza Bosilj, Nenad Pavin, Iva M. Tolić

Correspondence

npavin@phy.hr (N.P.),
tolic@irb.hr (I.M.T.)

In brief

The alignment of chromosomes at the spindle midplane is important for their accurate segregation. Risteski et al. link chromosome alignment with poleward flux by showing that longer kinetochore fibers slide faster than their shorter sister fibers. This tug-of-war between sister kinetochore fibers arises from lateral interactions with bridging microtubules.

Risteski et al., 2022, Cell Reports *40*, 111169
August 2, 2022 © 2022 The Author(s).
https://doi.org/10.1016/j.celrep.2022.111169

CellPress

Cell Reports

CellPress
OPEN ACCESS

Article

Length-dependent poleward flux of sister kinetochore fibers promotes chromosome alignment

Patrik Risteski,[1,3] Domagoj Božan,[2,3] Mihaela Jagrić,[1] Agneza Bosilj,[2] Nenad Pavin,[2,*] and Iva M. Tolić[1,4,*]
[1]Division of Molecular Biology, Ruđer Bošković Institute, Bijenička cesta 54, 10000 Zagreb, Croatia
[2]Department of Physics, Faculty of Science, University of Zagreb, Bijenička cesta 32, 10000 Zagreb, Croatia
[3]These authors contributed equally
[4]Lead contact
*Correspondence: npavin@phy.hr (N.P.), tolic@irb.hr (I.M.T.)
https://doi.org/10.1016/j.celrep.2022.111169

SUMMARY

Chromosome alignment at the spindle equator promotes proper chromosome segregation and depends on pulling forces exerted at kinetochore fiber tips together with polar ejection forces. However, kinetochore fibers are also subjected to forces driving their poleward flux. Here we introduce a flux-driven centering model that relies on flux generated by forces within the overlaps of bridging and kinetochore fibers. This centering mechanism works so that the longer kinetochore fiber fluxes faster than the shorter one, moving the kinetochores toward the center. We develop speckle microscopy in human spindles and confirm the key prediction that kinetochore fiber flux is length dependent. Kinetochores are better centered when overlaps are shorter and the kinetochore fiber flux slower than the bridging fiber flux. We identify Kif18A and Kif4A as overlap and flux regulators and NuMA as a fiber coupler. Thus, length-dependent sliding forces exerted by the bridging fiber onto kinetochore fibers support chromosome alignment.

INTRODUCTION

Chromosome alignment at the spindle equator in metaphase is a hallmark of mitosis and is important for proper completion of mitosis (Fonseca et al., 2019; Maiato et al., 2017). Chromosome movements on the spindle that lead to their alignment are driven by pulling forces exerted by kinetochore microtubules (kMTs) that pull the kinetochores poleward and polar ejection forces exerted by non-kMTs that push the chromosome arms away from the pole (Rieder and Salmon, 1994). The role of these forces in chromosome movements and alignment were explored in theoretical studies (Joglekar and Hunt, 2002; Civelekoglu-Scholey et al., 2006, 2013; Armond et al., 2015). The main mechanism of chromosome alignment in these models relies on polar ejection forces, which have a centering effect on chromosomes because these forces decrease away from the spindle pole (Ke et al., 2009).

Similarly to the polar ejection forces, pulling force generated by kMTs can have a centering effect on chromosomes even though forces generated at the microtubule (MT) plus end do not depend on MT length. The centering effect arises because of motor proteins such as kinesin-8, which "measure" MT length by binding along the MT lattice and walking all the way to the MT plus end, where they make MT dynamics length dependent (Varga et al., 2006). Indeed, kinesin-8 is required for chromosome alignment at the spindle center (Mayr et al., 2007; Stumpff et al., 2008, 2012; West et al., 2002). Theoretical studies have shown that length-dependent MT catastrophe induced by kinesins or length-dependent pulling forces can center kinetochores in yeast cells (Gardner et al., 2008; Mary et al., 2015; Gergely et al., 2016; Klemm et al., 2018). Thus, in addition to polar ejection forces, measuring of MT length by kinesins has an important contribution to chromosome centering.

However, this is not a complete picture of the forces that act on chromosomes. Kinetochore fibers (k-fibers) are also subjected to forces that drive their poleward flux (Forer, 1965; Hamaguchi et al., 1987; Hiramoto and Izutsu, 1977; Mitchison, 1989). This movement can be imagined as a conveyor belt-like transport whereby the whole k-fiber is shifted toward the pole, while its minus ends depolymerize and plus ends polymerize. This complex process is driven and regulated by multiple motor proteins (Miyamoto et al., 2004; Ganem et al., 2005; Rogers et al., 2004; Steblyanko et al., 2020). It has been proposed that poleward flux of k-fibers is generated by motor-driven sliding of k-fibers with respect to interpolar MTs (Mitchison, 2005), inspired by electron microscopy images of *Xenopus* extract spindles (Ohi et al., 2003). The mechanical interaction between k-fibers and the associated interpolar bundles called bridging fibers has been demonstrated by laser cutting of these fibers in human cells (Kajtez et al., 2016). Kinesin-5 activity contributes to the poleward flux of k-fibers and interpolar MTs in *Drosophila* syncytial embryo mitosis (Brust-Mascher et al., 2009). How poleward flux of interpolar MTs transmitted to k-fibers regulates forces acting on kinetochores has been explored in a theoretical model, which suggests that flux promotes tension uniformity on kinetochores, in agreement with experiments showing large variability in kinetochore tension in cells with abolished flux (Matos et al., 2009). Interestingly, physical coupling between k-fibers

This is an open access article under the CC BY license (http://creativecommons.org/licenses/by/4.0/).

and the associated interpolar bundles (bridging fibers) is important not only for tension but also for chromosome alignment, given that optogenetic perturbation of bridging fibers led to chromosome misalignment (Jagrić et al., 2021). In these experiments, chromosome misalignment was accompanied by elongation of bridging microtubule (bMT) overlaps. These recent findings, together with the idea that poleward flux is generated within bridging fibers and transmitted to k-fibers, open an interesting possibility that chromosome alignment, bMT overlaps and poleward flux are mutually related. Thus, the mechanism of chromosome alignment on the spindle is incompletely understood.

Here we hypothesize that poleward flux drives chromosome centering. We introduce a flux-driven centering model that relies on the interaction between bridging and k-fibers. The model describes a centering mechanism based on length-dependent pulling forces exerted by k-fibers onto the kinetochores. These forces increase with the overlap length between bridging and k-fibers and with the velocity difference between the fibers. To test this model, we developed a speckle microscopy assay on spindles of human cells, which allowed us to measure the flux of individual bMTs and kMTs. We found that at displaced kinetochores, the longer k-fiber undergoes flux at a higher velocity than the shorter one, which is at the core of the flux-driven centering because in this mechanism the faster flux of the longer k-fiber pulls the kinetochores in the direction of this fiber (i.e., toward the spindle center). Our experiments in which we performed a set of depletions of spindle proteins, together with theory, indicate that kinetochores are better centered when the overlaps between bridging and k-fibers are shorter and the k-fiber flux markedly slower than the bridging fiber flux. Forces from the bridging fiber are transmitted to the k-fiber in a manner dependent on the coupling between bridging and k-fibers. We show that k-fibers flux slower after depletion of NuMA, indicating that NuMA couples the fibers, whereas k-fibers flux faster after depletion of Kif18A (kinesin-8) and/or Kif4A (kinesin-4), which results in longer overlaps implying stronger coupling. Our results suggest that lateral length-dependent sliding forces that the bridging fiber exerts onto k-fibers promote the movement of kinetochores toward the spindle center.

RESULTS

Physical model for chromosome centering based on microtubule poleward flux

To explore the idea that MT poleward flux promotes kinetochore centering, we introduce a “flux-driven centering” model in which k-fibers laterally interact with bMTs (Figure 1A). The central idea of our theory is that kinetochores are centered by pulling forces proportional to the overlaps of k-fibers and bMTs. These forces are generated within the overlaps by the activity of motor proteins and by passive crosslinkers. When kinetochores are off-centered, the difference in the length of sister k-fibers leads to a difference in the length of antiparallel overlaps and thus of accumulated motors on either side, generating a centering force on the kinetochores (Figure 1A, white arrows). Similarly, a difference in the length of parallel overlaps and the number of accumulated crosslinkers also leads to centering of kinetochores (Figure 1A, gray arrows). Thus, the kinetochores become centered through tug-of-war between sister k-fibers, which is different from the previously proposed centering mechanism based on dynamics of k-fiber plus ends and polar ejection forces. By developing a theory for flux-driven centering, we explore how poleward flux centers kinetochores, and what aspects of the spindle are crucial for efficient centering.

A unique feature of our physical model is that motor proteins accumulate in the antiparallel overlaps between k-fibers and bMTs, where they slide the MTs apart. These sliding forces,

$$F_m = D n_m f_m, \quad \text{(Equation 1)}$$

are proportional to the overlap length, D, based on *in vitro* experiments (Shimamoto et al., 2015). The force is also proportional to the linear density of motors, n_m, each producing a force f_m. Parallel overlaps between k-fibers and bMTs are linked by passive crosslinkers, which help transmit the sliding forces from the bMTs to the k-fibers. Similar to the motor forces, the forces exerted by passive crosslinkers,

$$F_c = L n_c f_c, \quad \text{(Equation 2)}$$

are proportional to the length of parallel overlaps, L, the linear density of crosslinkers, n_c, and the force exerted by a single crosslinker, f_c. The forces generated by motors on a k-fiber are opposed by the force exerted at the kinetochore, F_{KC}, and by passive crosslinkers,

$$F_m = F_{KC} + F_c. \quad \text{(Equation 3)}$$

These are the main equations of the model, whereas a complete theory that includes not only the forces on k-fibers but also on bridging fibers, together with a force-velocity relationship for individual motors, and friction forces exerted by passive crosslinkers and kinetochores, is given in STAR Methods.

To explore the key features of the centering mechanism, we displace the kinetochores in the model by 2 μm away from the spindle center and explore how they return to the center (STAR Methods). The kinetochores approach the spindle center in several minutes for parameters typical for spindles in human (Figures 1B and 1C). When the kinetochores are displaced, the shorter k-fiber undergoes poleward flux at a slower velocity than the longer k-fiber, which is responsible for the movement of the kinetochores toward the spindle center (Figure 1D). The kinetochore centering velocity, which is equal to the half of the difference in poleward flux between two k-fibers, decreases as the kinetochores approach the center. The flux of both k-fibers is slower than the flux of bMTs (Figure 1D), making the centering mechanism work by allowing the k-fibers to slide at different velocities.

To study what features of the system are crucial for efficient centering, we test the dependence of the centering velocity and the k-fiber flux velocity on the model geometry, concentrations of motors and passive crosslinkers, and kinetochore parameters (Figures 1E–1H and S1A–S1D). We find that the length of antiparallel overlaps between bridging and k-fibers strongly affects the centering efficiency (Figures 1E and S1A–S1C). As the total antiparallel overlap between bridging and k-fibers

CellPress
OPEN ACCESS

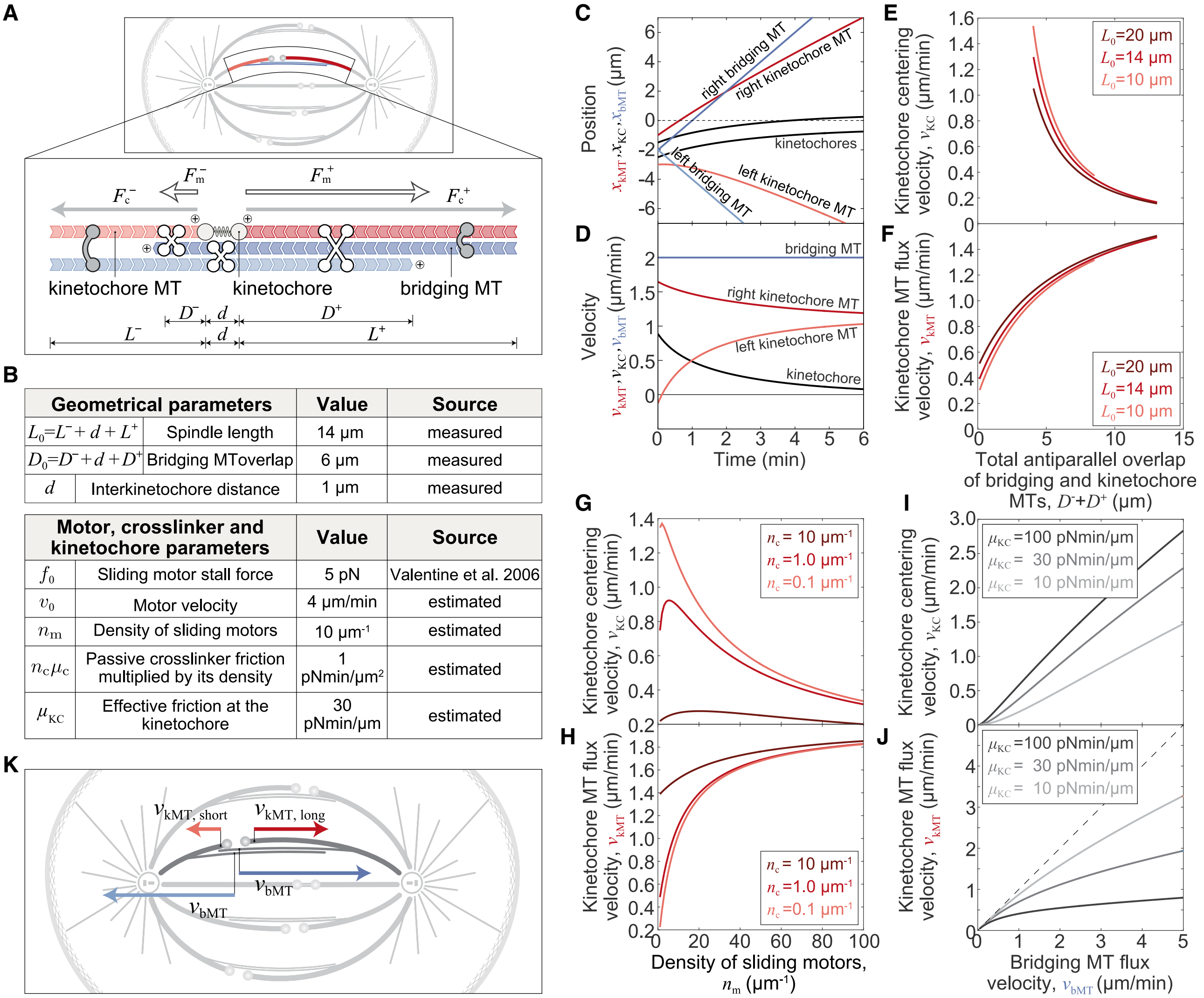

B

Geometrical parameters		Value	Source
$L_0=L^-+d+L^+$	Spindle length	14 μm	measured
$D_0=D^-+d+D^+$	Bridging MToverlap	6 μm	measured
d	Interkinetochore distance	1 μm	measured

Motor, crosslinker and kinetochore parameters		Value	Source
f_0	Sliding motor stall force	5 pN	Valentine et al. 2006
v_0	Motor velocity	4 μm/min	estimated
n_m	Density of sliding motors	10 μm^{-1}	estimated
$n_c\mu_c$	Passive crosslinker friction multiplied by its density	1 pNmin/μm^2	estimated
μ_{KC}	Effective friction at the kinetochore	30 pNmin/μm	estimated

Figure 1. Theoretical model for chromosome alignment

(A) Scheme of mitotic spindle (top) and the scheme of the model (bottom). KMTs (red) extend from the edges toward elastically connected kinetochores (spring connecting circles). BMTs (blue) extend from the edges toward each other. Motor proteins (white X-shapes) exert forces, $F_m^{\pm}$, between antiparallel MTs and passive crosslinkers (gray C-shapes) exert forces, $F_c^{\pm}$, between parallel MTs, where superscripts + and − denote the right and left sides, respectively.

(B) Parameters of the model.

(C and D) Solution of the model showing time course of positions (C) and velocities (D) of kinetochores (black), kMTs (red), and bMTs (blue) for kinetochores initially displaced 2 μm.

(E–J) Kinetochore centering velocities and kMT flux velocities for different values of (E and F) the length of antiparallel overlap and 3 values of spindle length, (G and H) sliding motor density and 3 values of passive crosslinker density, and (I and J) bMT flux velocity and 3 values of effective friction at the kinetochores. The dashed line in (J) denotes the case in which bMT and kMT flux velocities are equal.

(K) Scheme of mitotic spindle with flux velocities of kMTs (red arrows) and bMTs (blue arrows).

Parameters for all panels are given in (B) if not stated otherwise.

increases from 4 to 13 μm, the centering velocity decreases roughly 7-fold (Figure 1E). For the same increase of overlap length, the k-fiber flux velocity increases and consequently the difference between k-fiber and bridging fiber velocities decreases from 1.0 to 0.5 μm/min (Figure 1F). Centering is better for short overlaps because the relative difference in the number of motors on either side is larger, resulting in a greater centering velocity. Similarly, centering velocity decreases with decreasing spindle length, but the effect is smaller than for the overlap length (Figures 1E and 1F).

By varying the density of motor proteins, we find that the centering velocity has a maximum value below 20 motors/μm, around which the centering mechanism behaves optimally (Figures 1G and S1A). When the number of motors decreases from the optimum, the contribution of passive crosslinkers becomes larger than that of motors. This leads to worse centering

because passive crosslinkers generate smaller centering forces than motors. When the number of motors increases from the optimal one, the centering becomes worse for a different reason. Here, the k-fiber flux velocity increases (Figures 1H and S1A) and thus the difference between k-fiber and bridging fiber velocities decreases, because a large number of motors slide k-fibers poleward at a high velocity, leading to slower centering. Additionally, when the density of passive crosslinkers increases, leading to higher friction within parallel overlaps of bridging and k-fibers, the flux of k-fibers speeds up, and consequently centering is slower (Figures 1G, 1H, and S1B).

To explore the influence of the bridging fiber flux velocity on centering, we varied the velocity of motors in the absence of load, which is equal to the sliding velocity of the oppositely oriented bMTs with respect to one another or, in other words, twice the bMTs flux velocity. The kinetochore centering velocity and the k-fiber flux velocity increase with the bMTs flux velocity (Figures 1I and 1J). We also explored the influence of the effective friction at the kinetochore on centering and found that kinetochores center faster and k-fiber flux decreases for larger values of this parameter (Figures 1I, 1J, and S1C). The k-fiber flux is always slower than the bridging fiber flux (Figures S1A–S1C), and this difference is larger when the bridging fiber flux is faster (Figure 1J).

Taken together, the flux-driven centering model provides a crucial prediction that is unique to this model: at displaced kinetochores, the longer k-fiber undergoes flux at a higher velocity than the shorter one (Figures 1K; Video S1). The faster flux of the longer k-fiber pulls the kinetochores in the direction of this fiber (i.e., toward the spindle center). Thus, the difference in the flux of the sister k-fibers is the core of the centering mechanism.

Speckle microscopy assay to follow the movement of individual microtubules within the spindle

To test the predictions of the flux-driven centering model experimentally, it is important to measure the poleward flux of different classes of MTs (kinetochore and bridging), which requires analysis of the movements of individual MTs. Flux is typically studied by using tubulin photoactivation (Mitchison, 1989), a method in which all the MTs within the illuminated region are photoactivated, thus the movements of kMTs and non-kMTs cannot be distinguished. To overcome this issue, we developed an assay on the basis of speckle microscopy (Waterman-Storer et al., 1998) to study MTs within spindles of the human non-cancer immortalized epithelial cell line hTERT-RPE1 (hereafter referred to as RPE1). By using a very low concentration (1 nM) of SiR-tubulin (Lukinavičius et al., 2014), we obtained speckled signal of SiR-tubulin in the spindle (Figure 2A; Video S2), which comes from a few dye molecules within a resolution-limited region (Waterman-Storer and Salmon, 1998).

To identify the speckles that are localized on kMTs or bMTs, we follow the position of their first appearance and their subsequent movement. The speckles that originate close to a kinetochore, at the pole-facing side, were defined as those on a kMT (Figure 2B). The speckles that appear on one side of a pair of sister kinetochores, pass the region between them, and end up on the other side, were defined as those on a bMT (Figure 2C). All other speckles in the spindle region between the centrosomes, for which we cannot determine the type of MT they belong to, we refer to as "other" speckles (Figures S2A and S2B; see STAR Methods). We tracked individual speckles (Figure S2C) together with the spindle poles marked by centrioles and calculated poleward flux as the change of the speckle-to-pole distance over the first 30 s of their movement (Table 1). This assay allowed us to study the movement of kMTs and bMTs with respect to the poles and to each other.

To explore the relevance of the model to kinetochore alignment, we used this assay in unperturbed cells and after a set of perturbations in which we depleted candidate MT-associated proteins by small interfering RNA (siRNA). We depleted motor proteins that are known to be involved in kinetochore alignment and/or localize to the bridging fiber (Kif18A, Kif4A, Kid, CENP-E, and MKLP1) and non-motor proteins that are important for k-fiber and bridging fiber integrity and their crosslinking (PRC1, Haus8, and NuMA) (Maiato et al., 2017; Pavin and Tolić, 2021). For all these treatments, we analyzed the poleward flux of bridging and k-fibers, kinetochore positions, and the length of antiparallel overlaps (Table 1). Although these treatments most likely also affect other aspects of the spindle architecture and dynamics, we expect to identify general interdependence between flux dynamics and kinetochore centering.

Longer kinetochore fiber undergoes flux at a higher velocity than the shorter one

The central prediction of the flux-driven centering model is that kinetochore centering relies on a difference in the flux velocity of sister k-fibers, where the flux of the longer k-fiber is faster than the flux of the shorter one. To explore whether this prediction holds in real spindles, we compared the flux of k-fibers of different lengths by using our speckle microscopy assay. The speckles on k-fibers, defined as those originating close to a kinetochore, were located at various distances from the pole, which correspond to the k-fiber length.

Strikingly, the k-fiber poleward velocity increased with an increasing k-fiber length in untreated cells ($p = 4e-04$, $n = 164$; Figures 2D–2F). The same trend was observed when the k-fibers were divided into 3 groups, short, medium, and long, as those with lengths smaller than 0.4, between 0.4 and 0.6, and larger than 0.6 of the spindle length, respectively. Short k-fibers had a flux of 0.91 ± 0.08 μm/min ($n = 51$ speckles from 68 cells), whereas the flux of long k-fibers was significantly faster, 1.81 ± 0.34 μm/min ($n = 11$ speckles from 68 cells, $p = 3e-04$), and the flux of medium k-fibers was between these values. The average poleward flux velocity of all speckles on k-fibers was 1.23 ± 0.06 μm/min ($n = 164$ speckles from 68 cells), which is similar to the flux rate previously measured by tubulin photoactivation on k-fibers in RPE1 cells expressing photoactivatable-GFP-α-tubulin (Dudka et al., 2018), supporting our criteria for identification of speckles on k-fibers.

To test the difference in flux between short and long k-fibers by an independent method, we used photoactivation assay on U2OS cells with stable expression of photoactivatable-GFP-α-tubulin (Figures 2G and S2D). By sequentially photoactivating sister k-fibers of an individual kinetochore pair found outside the metaphase plate during its oscillations, we found that the longer

CellPress
OPEN ACCESS

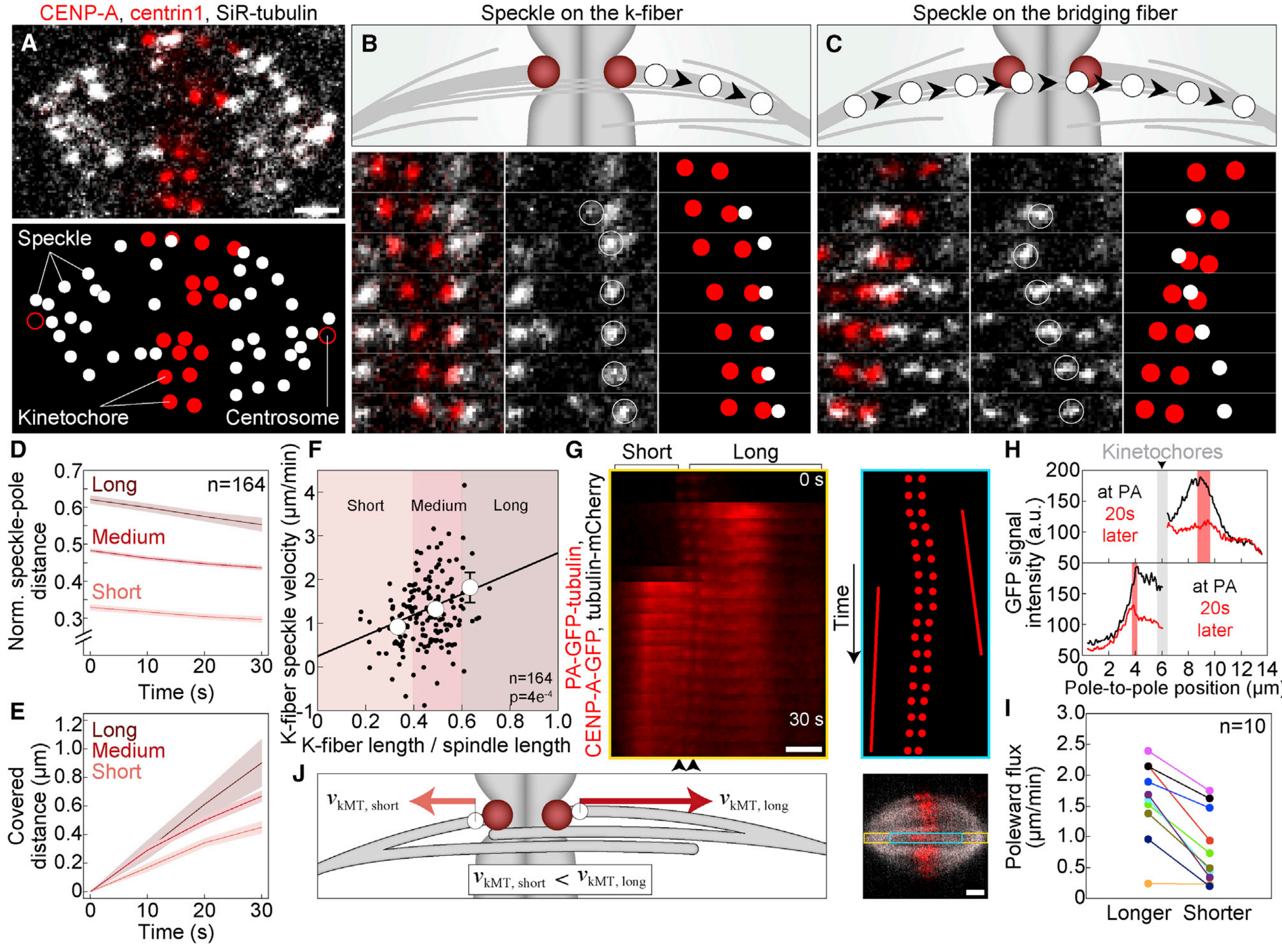

Figure 2. Poleward flux promotes kinetochore movement toward the spindle midplane

(A–C) Speckle microscopy assay for measurement of poleward flux of individual MTs. (A) Spindle in a RPE1 cell stably expressing CENP-A-GFP and centrin1-GFP (red) stained with 1 nM SiR-tubulin dye, which appears as distinct speckles marking individual MTs (gray). (B) Scheme of a speckle originating at the kinetochore defined as the one marking a kMT (top). Montage over time demonstrating the movement of the speckle belonging to the kMT (bottom). Left shows merge, middle shows SiR-tubulin channel with encircled speckle, and right shows schematic of kinetochores (red) and speckle (white) positions. (C) Scheme of a speckle passing the region between sister kinetochores, moving close to the kinetochores, defined as the one marking a MT within the bridging fiber (top). Montage over time demonstrating the movement of the speckle belonging to the bridging MT. Legend as in (B).

(D) Speckle-pole distance over time divided by spindle length for k-fibers classified as short, medium, and long, according to the k-fiber length being smaller than 0.4, between 0.4 and 0.6, and larger than 0.6 of the spindle length, respectively. Lines, mean; shaded areas, SEM.

(E) Change in speckle-pole distance over time for speckles within groups as in (D). Lines, mean; shaded areas, SEM.

(F) Poleward velocity of k-fiber speckles within groups as in (D) depending on its relative starting speckle-pole distance. Circles, mean; error bars, SEM.

(G) Montage over time (left) and scheme (right) of a photoactivated region in U2OS cell (bottom) stably co-expressing PA-GFP-α-tubulin (red), CENP-A-GFP (red), and mCherry-α-tubulin (gray). Time interval, 2 s. Shorter and longer sister k-fiber and kinetochore positions (black arrows) are shown. In scheme (right), lines highlight poleward motion of the photoactivated regions.

(H) Graphs show pole-to-kinetochore profile intensities of GFP signal for longer (top) and shorter (bottom) k-fiber from spindle in G at the time of photoactivation (black line) and 20 s later (red line). Red shaded areas, covered distance of photoactivated regions; gray shaded areas, kinetochore positions.

(I) Poleward flux of longer and shorter sister k-fiber retrieved from photoactivation assay in U2OS cells and color-coded for each pair.

(J) Scheme of speckles on longer and shorter k-fiber, where the speckle on the longer k-fiber fluxes faster than the speckle on the shorter k-fiber. Scale bars, 2 μm.

sister k-fiber fluxes faster than the corresponding shorter sister k-fiber (Figures 2H and 2I). Overall, the poleward flux increased with an increasing k-fiber length (Figure S2E). Thus, our experiments on the basis of two independent methods, speckles and photoactivation, reveal that longer k-fibers flux faster than shorter ones, which is a key feature of the flux-driven centering mechanism (Figure 2J).

Bridging microtubules undergo poleward flux at a higher velocity than kinetochore microtubules

In the flux-driven centering mechanism, motors within the bridging fiber drive the flux of bMTs, and the interaction between the bridging and k-fibers generates the flux of k-fibers. However, the tension between sister kinetochores opposes the flux of k-fibers, making it slower than the flux of the bridging fiber. This

Table 1. Measurements of flux, spindle, and kinetochore parameters

	Flux, all (μm/min)	Flux, k-fiber (μm/min)	Flux, bridge (μm/min)	Flux, other (μm/min)	Spindle length (μm)	Overlap length (μm)	Kinetochore distance to equatorial plane (μm)
Untreated	1.27 ± 0.05 (371, 68, NA)	1.23 ± 0.06 (164, 68, NA)	2.07 ± 0.11 (101, 68, NA)	0.56 ± 0.09 (106, 27, NA)	13.87 ± 0.23 (44, NA)	6.6 ± 0.2 (33, 11, NA)	0.98 ± 0.05 (258, 44, NA)
Kif18A	1.68 ± 0.12 (119, 27, 0.002)	1.72 ± 0.18 (52, 27, 0.01)	2.07 ± 0.23 (37, 27, 0.9)	1.13 ± 0.22 (30, 21, 0.02)	15.12 ± 0.33 (25, 0.003)	8.1 ± 0.3 (35, 14, 1e-04)	1.50 ± 0.08 (198, 28, 9e-6)
Kif4A	1.66 ± 0.08 (132, 30, 1e-04)	1.85 ± 0.11 (57, 30, 3e-06)	2.14 ± 0.15 (36, 30, 0.6)	0.96 ± 0.14 (39, 10, 0.01)	15.70 ± 0.28 (25, 7e-06)	7.4 ± 0.2 (39, 10, 6e-04)	0.93 ± 0.06 (165, 25, 0.4)
Kid	1.52 ± 0.08 (106, 24, 0.01)	1.32 ± 0.10 (51, 24, 0.4)	2.06 ± 0.15 (33, 24, 0.9)	1.15 ± 0.12 (22, 7, 1e-04)	13.29 ± 0.69 (10, 0.4)	6.6 ± 0.2 (33, 12, 0.9)	1.03 ± 0.14 (57, 10, 0.5)
CENP-E	0.59 ± 0.07 (70, 9, 7e-13)	0.55 ± 0.14 (22, 9, 8e-05)	0.91 ± 0.15 (17, 9, 5e-07)	0.44 ± 0.07 (31, 9, 0.2)	15.17 ± 0.23 (20, 2e-04)	6.3 ± 0.2 (28, 11, 0.17)	0.66 ± 0.05 (122, 25, 1e-04)
MKLP1	1.08 ± 0.09 (78, 13, 0.07)	0.93 ± 0.13 (34, 13, 0.03)	1.43 ± 0.20 (21, 13, 0.007)	0.99 ± 0.14 (23, 13, 0.01)	13.39 ± 0.39 (10, 0.3)	6.9 ± 0.1 (32, 14, 0.2)	0.76 ± 0.09 (57, 10, 0.02)
PRC1	1.32 ± 0.08 (145, 28, 0.5)	1.34 ± 0.10 (79, 28, 0.3)	2.23 ± 0.15 (29, 28, 0.3)	0.57 ± 0.10 (37, 11, 0.9)	13.86 ± 0.24 (15, 0.97)	NA	0.73 ± 0.05 (93, 15, 0.01)
Haus8	0.79 ± 0.06 (175, 34, 9e-09)	0.71 ± 0.07 (87, 34, 4e-08)	1.35 ± 0.18 (39, 34, 9e-04)	0.50 ± 0.06 (49, 14, 0.5)	13.46 ± 0.38 (23, 0.36)	6.5 ± 0.1 (30, 13, 0.7)	0.84 ± 0.06 (137, 23, 0.09)
NuMA	0.95 ± 0.08 (157, 32, 8e-04)	0.78 ± 0.09 (53, 32, 9e-05)	2.03 ± 0.16 (38, 32, 0.8)	0.45 ± 0.08 (66, 13, 0.3)	14.42 ± 0.32 (17, 0.18)	6.8 ± 0.1 (33, 10, 0.45)	0.96 ± 0.07 (130, 17, 0.7)
Kif18A + Kif4A	1.82 ± 0.12 (105, 23, 3e-05)	1.92 ± 0.20 (43, 23, 0.002)	1.92 ± 0.21 (36, 23, 0.5)	1.50 ± 0.15 (26, 23, 3e-06)	16.96 ± 0.31 (37, 1e-11)	8 ± 0.2 (35, 13, 1e-04)	2.86 ± 0.12 (235, 37, 2e-16)
Kif18A + PRC1	1.91 ± 0.10 (134, 16, 8e-08)	2.00 ± 0.15 (70, 16, 3e-06)	2.18 ± 0.23 (32, 16, 0.6)	1.42 ± 0.13 (32, 16, 5e-07)	15.41 ± 0.44 (18, 0.005)	NA	2.19 ± 0.15 (116, 18, 8e-15)
Kif18A + Haus8	0.98 ± 0.11 (90, 30, 0.01)	0.71 ± 0.11 (60, 30, 1e-04)	1.59 ± 0.20 (30, 30, 0.01)	ND	ND	ND	1.20 ± 0.05 (242, 30, 0.002)
Kif18A + Kif4A + PRC1	1.59 ± 0.10 (98, 16, 0.005)	1.79 ± 0.16 (43, 16, 0.001)	2.02 ± 0.27 (18, 16, 0.8)	1.15 ± 0.11 (37, 16, 7e-05)	17.09 ± 0.29 (20, 4e-11)	NA	3.20 ± 0.18 (91, 20, 2e-16)
Ndc80	1.71 ± 0.16 (44, 8, 0.01)	NA	1.97 ± 0.22 (25, 8, 0.6)	1.36 ± 0.22 (19, 8, 0.002)	15.09 ± 0.29 (15, 0.002)	ND	1.38 ± 0.11 (105, 15, 0.003)

Values are given as mean ± SEM. The numbers in parentheses denote the number of measurements (number of speckles for flux measurements or number of kinetochore pairs; for spindle length this number is not given, because it is equal to the number of cells), number of cells, and p value from a t test or Mann-Whitney test (last column) for comparison with untreated cells. Results for k-fiber flux velocity after depletions of Kid, PRC1, NuMA, and bridging fiber flux in Ndc80 are in agreement with Steblyanko et al. (2020), except for Kif4A.
NA, not applicable; ND, not determined.

difference between the bridging and k-fiber flux is found in the model for various parameters (Figures 1D, 1F, 1H, and 1J), so we asked if the same feature is also observed in experiments.

Remarkably, speckles on the bMTs moved poleward at a velocity of 2.07 ± 0.11 μm/min in untreated cells (n = 101 speckles from 68 cells), which is significantly faster than for the speckles on kMTs (p = 1e-10) (Figures 3A–3D; Table 1). In contrast to k-fibers, bridging fiber flux did not depend on the position of the associated kinetochores along the spindle axis (Figure S3A), additionally supporting the result that this dependence is k-fiber specific.

Because our experiments provide the first measurement of poleward flux of bMTs in human spindles, we decided to validate our method of identification of speckles in the bridging fiber. First, the distance between these speckles and the kinetochore-kinetochore axis of the associated k-fibers was 0.15 ± 0.01 μm, which was similar to the previously measured bridge-kinetochore distance (Kajtez et al., 2016; Polak et al., 2017) and significantly smaller than the distance to the kinetochore-kinetochore axis of their nearest neighbors, 0.89 ± 0.04 μm (n = 101, p = 2e-16; Figure S3B). Furthermore, we used PRC1 siRNA, which is known to specifically reduce the number of bMT to ~50% of the original number (Jagrić et al., 2021; Polak et al., 2017). In agreement with this, cells treated with PRC1 siRNA had a roughly 2-fold smaller ratio of bMT to kMT speckles in comparison with untreated cells, providing support for our method of identification of speckles on bridging fibers (0.37 ± 0.05 versus 0.62 ± 0.04; Figure S3C).

The observed rate of bMT poleward flux implies that the antiparallel bMTs slide apart with respect to each other at twice the rate of their poleward flux (i.e., 4.1 ± 0.2 μm/min), given that the spindle length is constant during metaphase. This rate is comparable with the sliding rate of bMTs in early anaphase measured by tubulin photoactivation, which is roughly 4.5 μm/min (Vukušić et al., 2021), suggesting that the bMT sliding may be driven by a similar mechanism in metaphase and early anaphase.

CellPress
OPEN ACCESS

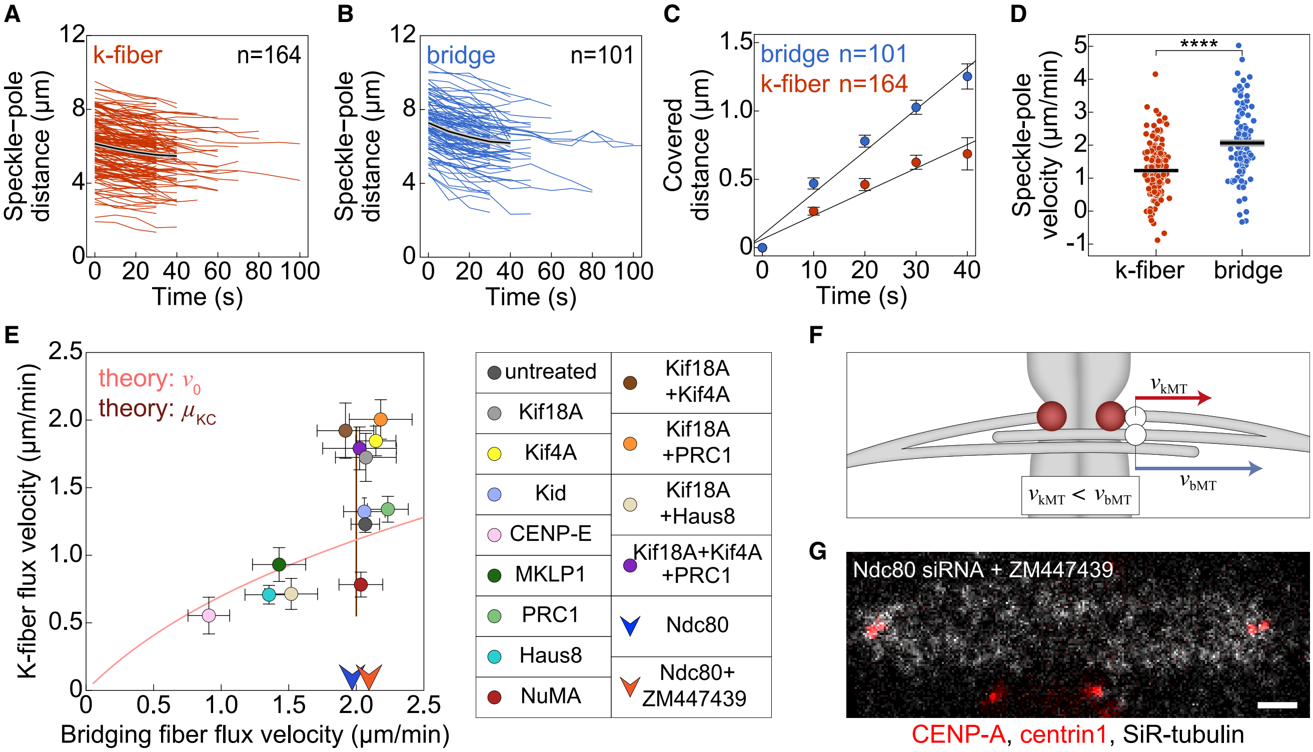

Figure 3. Bridging microtubules flux faster than kinetochore microtubules

(A and B) Distance between kMT (A) and bMT (B) speckles from the corresponding pole over time in untreated cells. Colored lines show individual speckles. Black line, mean; gray area, SEM.

(C) Change in speckle-pole distance over time for speckles within k-fibers and bridging fibers in untreated cells. Circles, mean; error bars, SEM.

(D) Poleward velocity of the k-fiber and bridging fiber speckles. Each dot corresponds to an individual speckle. Black lines, mean; gray areas, SEM.

(E) Poleward velocity of the k-fiber versus poleward velocity of the bridging fiber. Circles, mean; error bars, SEM. siRNA treatments are color-coded; see legend. Note that Ndc80-depleted and Ndc80-depleted and ZM447439-treated cells are shown as arrows because poleward velocity of k-fibers could not be assessed. Theoretical predictions (lines) for v_0 = 0.1–10 µm/min (pink), and for μ_{KC} = 1–100 pNmin/µm (brown), x_{KC} = 0 µm, and other parameters are as in Figure 1B.

(F) Scheme showing that a speckle within the bridging fiber fluxes faster than a speckle within the k-fiber.

(G) Spindle in a cell treated with Ndc80 siRNA and ZM447439 inhibitor. Legend as in Figure 2A. Scale bar, 2 µm.

Statistical analysis conducted using t test. *p = 0.01–0.05, **p = 0.01–0.001, ***p = 0.001–0.0001, ****p < 0.0001; ns, p ≥ 0.05.

To explore the relationship between the bridging and k-fiber flux under various perturbations of the spindle, we measured the flux after a set of depletions of spindle proteins given in Table 1 (see Figures S4A–S4I for depletion efficiency and Figures S5A–S5M for all speckle velocities). Strikingly, the flux of bridging fibers was faster than or equal to the flux of k-fibers across the treatments, even though the relationship between these two velocities was complex (Figures 3E and 3F). To compare these data with theoretical predictions, we first varied the motor velocity and found that the model prediction explains the data points with slower bridging fiber flux (Figure 3E). For the treatments that had unchanged bridging fiber flux, we varied the effective friction on the kinetochore because an increase in this friction slows down the k-fiber flux and vice versa in the model (Figure S1C), which agreed with this subset of treatments (Figure 3E). Thus, our model together with experiments suggests that the used treatments can be divided roughly into two groups, in one of which the sliding velocity was altered, whereas in the other the interaction between the k-fiber and kinetochore. Because faster bridging than k-fiber flux is a signature of the flux-driven centering mechanism, our experimental findings over various treatments suggest that the bridging fiber flux drives the k-fiber flux.

To explore whether bMTs are at the origin of the differential k-fiber flux in longer and shorter k-fibers, we tested the relative flux distribution in treatments which perturb the number of MTs in the bridging fiber. We found that k-fibers in PRC1-depleted spindles undergo similar differential flux as in untreated ones, whereas Haus8-depleted spindles showed no differential k-fiber flux rates (Figure S6A). This is in agreement with the fact that Haus8 depletion perturbs bridging fibers to a larger extent than PRC1 (Jagrić et al., 2021; Manenica et al., 2020).

To study to what extent k-fibers affect the sliding of bMTs, we depleted Ndc80, the main coupler of kinetochores to MT ends (Cheeseman et al., 2006; Cheeseman and Desai, 2008) (Figures 3E and S6B; Video S3). As expected, we did not detect speckles on k-fibers (i.e., those at the pole-facing side of the kinetochore) after Ndc80 depletion (n = 8 cells). We found that the speckles on bMTs fluxed at a similar velocity as in untreated cells (Figure 3E; Table 1), suggesting that sliding of bMTs is largely unaffected by k-fibers and that the poleward flux is

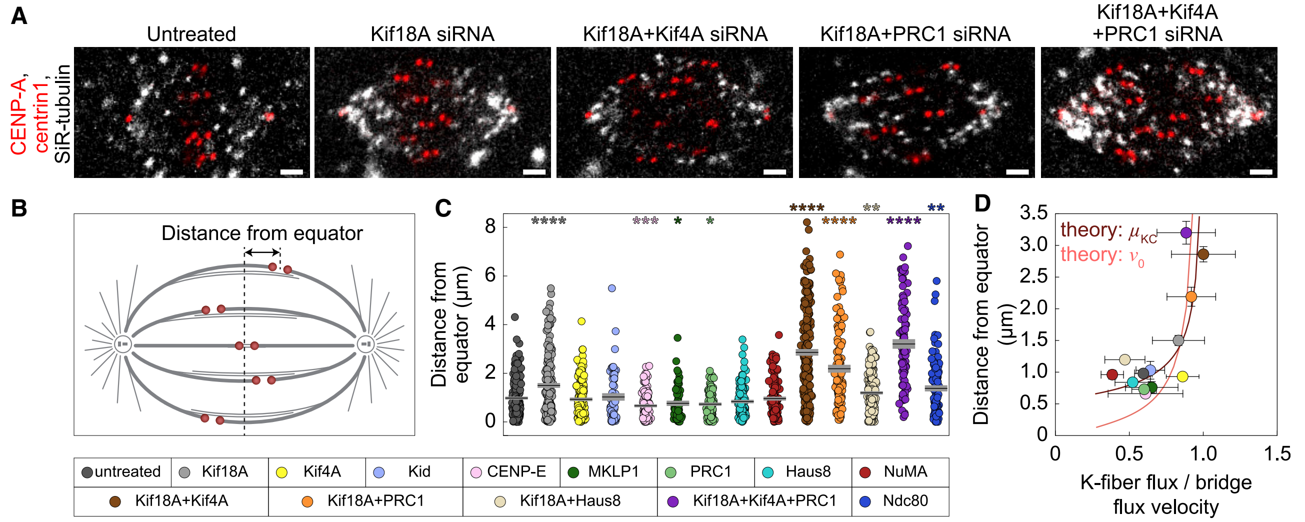

Figure 4. Kinetochore alignment depends on the ratio of k-fiber to bridging fiber flux velocity

(A) Spindles in untreated, Kif18A, Kif18A and Kif4A, Kif18A and PRC1, and Kif18A, Kif4A and PRC1 depleted cells (from left to right). Legend as in Figure 2A. Scale bars, 2 μm.

(B) Scheme shows that the distance from equator was measured as the distance between sister kinetochore midpoint and the equatorial plane.

(C) Kinetochore distance from equator in untreated and siRNA-treated cells. Each treatment is compared with untreated cells. Black lines, mean; gray areas, SEM.

(D) Experimental data for the kinetochore distance from equator versus ratio of k-fiber to bridging fiber flux velocity in untreated and siRNA-treated cells. Circles; mean. Error bars; SEM. Theoretical predictions for centering efficiency, described as $x^2 = 2DT$, where T is centering time and is calculated from kinetochore distance from center and centering velocity, $T = x_{KC}/v_{KC}$, as a function of the ratio of k-fiber to bridging fiber flux velocities. $D = 0.009$ μm^2/min and 0.1 μm^2/min, obtained from the fit to the data by varying the model parameter motor velocity (pink curve) or the effective friction at the kinetochore (brown curve), respectively. Treatments in (C) and (D) are color-coded according to the legend at the bottom. Statistical analysis was conducted using the Mann-Whitney test; p values as in Figure 3.

generated within the bridging fiber. Moreover, we found this velocity to be similar to MT poleward flux in the spindles without k-fibers and lateral kinetochore attachments to the spindle obtained by Ndc80 depletion and Aurora B inhibition by ZM447439 (Figures 3E and 3G). By perturbing a set of proteins, we were unable to increase the rate of bridging fiber flux in the spindles, which suggests that the bMTs flux at their maximal rate. However, in treatments where bridging fiber flux was reduced, because of Haus8, CENP-E, or MKLP1 depletion, k-fiber flux velocities were also reduced (Figures 3E and S6C; Table 1), suggesting that these proteins affect antiparallel sliding within bridging fiber overlaps and consequently k-fiber sliding.

Kinetochore centering efficiency depends on the flux velocity of k-fibers

At the core of this centering mechanism is that the shorter k-fiber has slower flux than the longer sister k-fiber, generating a flux difference that moves the off-centered kinetochores toward the spindle center. This difference in flux requires the average k-fiber flux to be slower than the bridging fiber flux. Thus, centering is more efficient when k-fiber flux is slower than the bridging fiber flux, allowing sliding of k-fibers along bridging fibers, which provides an important testable prediction of the model (Figures 1E–1J).

To compare our experiments with the model, we quantified kinetochore centering efficiency by measuring the distances of sister kinetochore midpoints from the equatorial plane of the spindle (Figures 4A–4C; Video S4) and explored how this distance depends on the ratio of the k-fiber to bridging fiber flux velocities across all treatments (Figures 4D and S7A). The treatments with this ratio similar to or lower than that of untreated cells show efficient centering comparable with untreated cells. In contrast, treatments with larger ratio of k-fiber to bridging fiber flux velocities show worse centering, except Kif4A depletion, which we comment on in the discussion. Worse centering with respect to untreated cells was found only in treatments that included Kif18A depletion (Figure 4C and Table 1; note that Ndc80 depletion resulted in worse centering because of abolished k-fibers). As Kif18A has a major role in k-fiber plus-end dynamics and thus in kinetochore alignment (Stumpff et al., 2008), it is important to test the link between flux and kinetochore alignment independently of Kif18A. Thus, we focus on the treatments that include Kif18A depletion to decouple flux-driven centering mechanism from the role of Kif18A in k-fiber tip regulation. Among the five treatments where Kif18A was depleted, co-depletion of Haus8 and Kif18A resulted in the lowest ratio of the k-fiber to bridging fiber flux and best kinetochore alignment (Figure 4D). In contrast, Kif18A depletion alone and co-depletions with Kif4A, PRC1, or Kif4A and PRC1 resulted in a high flux ratio, which was not different from 1 ($p > 0.34$ for each treatment). In these four treatments with high flux ratio, kinetochore alignment was worse than in Haus8/Kif18A co-depletion ($p < 0.02$ for each of the four treatments, Mann-Whitney test). Thus, flux ratio is related to kinetochore alignment in Kif18A-depleted background,

CellPress
OPEN ACCESS

suggesting that the effect of flux-driven centering can be observed in the absence of Kif18A-dependent k-fiber plus-end dynamics.

Interestingly, predictions from the model obtained in two different ways, by varying either the effective kinetochore friction or the motor velocity, showed a trend similar to the experimental data, even though each experimental treatment likely altered several spindle features (Figure 4D). The model prediction with varying the effective kinetochore friction agrees more closely with the experimental data than the one with varying motor velocity likely because among the used treatments, many of them altered the dynamics of the k-fiber plus end, such as those that include Kif18A depletion, whereas only a few treatments changed the sliding velocity. Thus, the experiments together with theory suggest that the ratio of k-fiber to bridging fiber flux velocities influences chromosome alignment.

As a control for proper attachment of misaligned kinetochores we imaged astrin, which binds to end-on attached kinetochores (Shrestha et al., 2017), and found it localized at all kinetochores including those that were highly off-centered (Figure S7B). This suggests that the reason for off-centering was not lack of kinetochore biorientation. We also note that the observed worse centering after combined depletion of Kif18A and Kif4A in comparison with Kif18A depletion differs from a previous study (Stumpff et al., 2012). This difference is not due to the use of different cell lines, as we obtained similar results on HeLa and U2OS cells as on RPE1 (Figures S7C and S7D), but likely related to a different effect of the double depletion on spindle length. Taken together, our experiments and the model suggest that kinetochores are better centered when the k-fiber flux is markedly slower than the bridging fiber flux, allowing sliding of k-fibers along bridging fibers and thus the movement of the center of sister k-fibers toward the spindle center.

Longer overlaps of antiparallel microtubules lead to an increase in the k-fiber flux velocity to the bridging fiber flux velocity

Our experiments have shown that an increased flux velocity of k-fibers is related to less efficient kinetochore centering. What caused this speeding up of the k-fiber flux in the treatments with misaligned kinetochores? The model suggests that changes in the overlap length can lead to changes in flux velocities (Figure 1F), even though these two quantities are not obviously correlated.

To explore this intriguing relationship, we measured the overlap length by measuring the length of PRC1-labeled regions in all the treatments except those where PRC1 was depleted (Figures 5A, 5B, and S7E). Among these treatments, overlaps were longer after depletion of Kif18A or Kif4A, in agreement with previous results (Jagrić et al., 2021), and after a combined depletion of Kif18A and Kif4A (Table 1; Figure S7F; see Figure S7G for HeLa and U2OS cells). These treatments specifically increased the flux velocity of k-fibers without changing the flux of bridging fibers, resulting in k-fibers fluxing at ~90% of the bridging fiber flux velocity (Figures 5C and 5D; Table 1). For comparison, in untreated cells, k-fibers flux at ~60% of the bridging fiber flux velocity (Table 1). Thus, these experiments reveal a relationship between the overlap length and the k-fiber flux velocity, suggesting that the sliding forces generated within the bridging fiber are transferred to the k-fibers through the antiparallel overlaps between these two types of fibers.

The difference in the flux of bridging and k-fibers increases for smaller concentrations of passive crosslinkers

The sliding forces from the bridging fiber are transmitted to the k-fibers not only through the antiparallel overlaps but also through the regions of parallel overlaps, where the bMTs and kMTs extending from the same spindle half are linked together by passive crosslinkers (see Figure 1A). Thus, reducing the amount of passive crosslinkers should result in reduced force transmitted from the bridging to the k-fibers and consequently in slower flux of k-fibers, as predicted by the model (Figure 1H).

To explore the role of passive crosslinkers in the parallel overlaps of bMTs and kMTs, we chose NuMA as a candidate because it is required for local load bearing in the spindle (Elting et al., 2017) (Figure 5E) and for synchronous MT flux across the spindle (Steblyanko et al., 2020). After depletion of NuMA by siRNA (Figures 5F and S4H; Video S5), we found that the flux velocity of kMTs decreased by ~40% (from 1.23 ± 0.06 μm/min in untreated cells to 0.78 ± 0.09 μm/min after NuMA depletion; Figures 5G, 5H, and S5H; Table 1). On the contrary, the flux velocity of bridging fibers did not change significantly (Figures 5G and 5H); thus the difference compared with the k-fiber velocity increased. Because the model predicts a larger difference in flux velocities for fewer passive crosslinkers (Figure 1H), these results support the idea that NuMA acts as a passive crosslinker transmitting the sliding forces from the bridging fiber onto the associated k-fibers through their parallel overlaps.

DISCUSSION

On the basis of our model and the results from speckle microscopy that allowed us to measure the relative movements of kMTs and bMTs, we propose that MT poleward flux promotes kinetochore centering because the flux of a longer sister k-fiber is faster than the flux of the shorter one, resulting in the kinetochores movement toward the spindle center (Figure 6A). The efficiency of kinetochore centering depends on the length of overlaps between bMTs and kMTs (Figure 6B).

The flux-driven centering mechanism proposed here and the previously introduced centering forces based on length-dependent suppression of k-fiber dynamics (Gardner et al., 2008; Mary et al., 2015; Gergely et al., 2016; Klemm et al., 2018) as well as on polar ejection forces (Joglekar and Hunt, 2002; Civelekoglu-Scholey et al., 2006, 2013; Armond et al., 2015) are conceptually independent. Yet proteins such as Kif18A and Kif4A may be involved in more than one mechanism: in addition to their role in regulating bMT overlap (Jagrić et al., 2021), Kif18A regulates k-fiber dynamics (Stumpff et al., 2008) and Kif4A is a chromokinesin that affects the flux by pushing on chromosome arms (Steblyanko et al., 2020). Diverse centering mechanisms may work together but with different efficiency

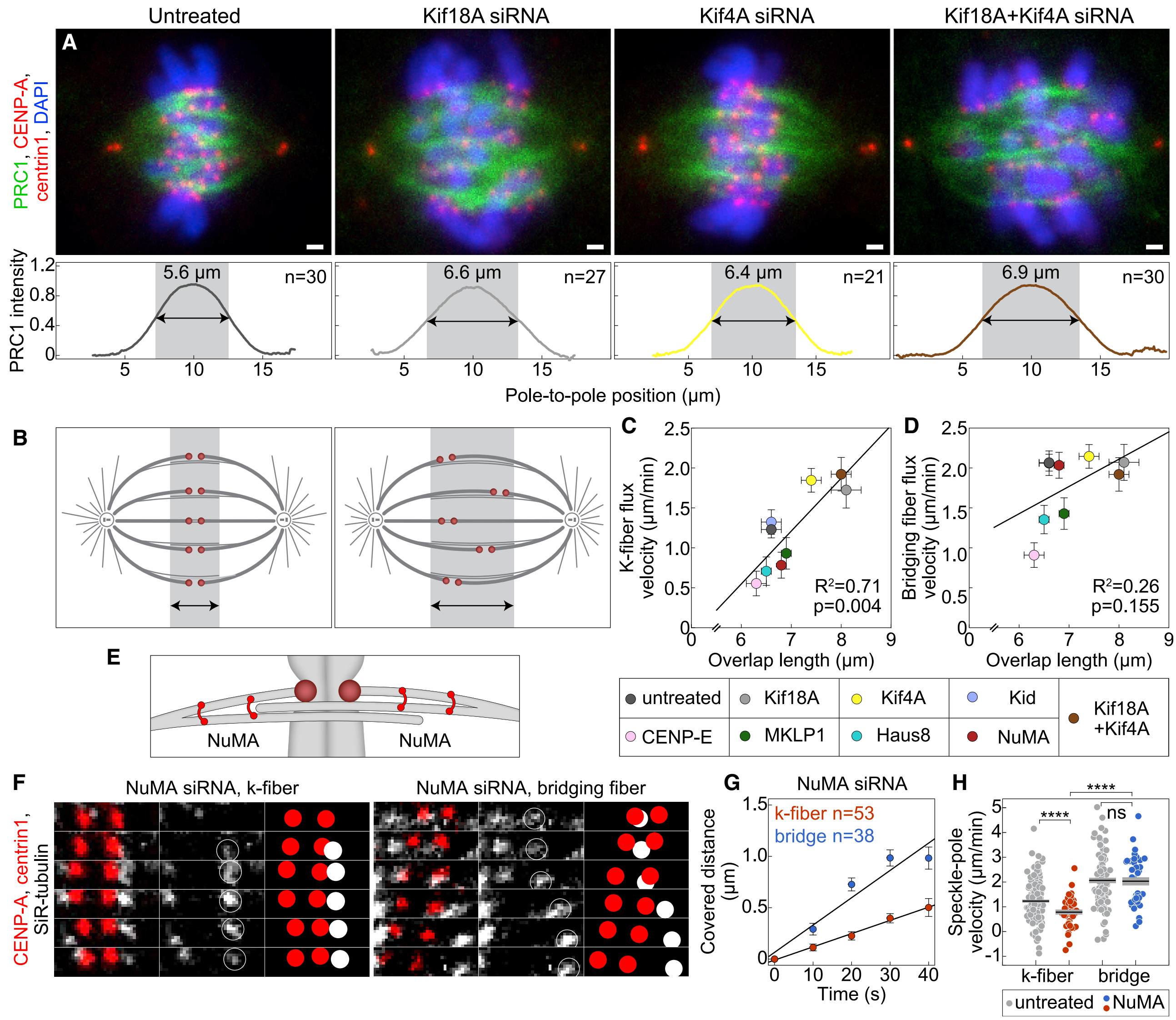

Figure 5. Coupling between bridging and k-fibers controls k-fiber flux velocity

(A) Fixed spindles in RPE1 cells stably expressing CENP-A-GFP and centrin1-GFP (red). Cells are untreated, Kif18A, Kif4A, and Kif18A and Kif4A depleted (from left to right), immunostained for endogenous PRC1 (AF-594, green) and stained with DAPI (blue). Images are sum intensity projections of five z-planes. Scale bars, 1 μm. Graphs show normalized pole-to-pole PRC1 intensity profiles of complete spindles for corresponding treatments. For individual cells see Figure S7E. Colored line, mean.

(B) Scheme shows that spindles with shorter (left) and longer (right) overlap regions have better (left) and worse (right) kinetochore alignment at the spindle equator, respectively.

(C and D) K-fiber (C) and bridging fiber (D) flux velocity versus PRC1-labeled overlap length. Treatments are color-coded as shown in the legend below. Circles, mean; error bars, SEM.

(E) Scheme of NuMA localization.

(F) Montage over time demonstrating the movement of a speckle belonging to the k-fiber (left) and bridging fiber (right) in NuMA siRNA treatment. Legend as in Figure 2B.

(G) Change in speckle-pole distance over time for speckles within bridging and k-fibers in cells treated with NuMA siRNA. Circles, mean; error bars, SEM.

(H) Poleward velocity of the speckles in NuMA siRNA-treated (red, k-fiber; blue, bridging fiber) and untreated (gray) cells. Black lines, mean; gray areas, SEM. Statistical analysis was conducted using t test; p values as in Figure 3.

depending on the cell type and the stage of spindle assembly. Because of the complexity of the spindle, it is hard to dissect the contribution of different mechanisms by using only experimental approaches (Tolić and Pavin, 2021), but future theoretical studies that would include multiple MTs, regulation of their plus-end dynamics, MT nucleation along pre-existing MTs, and polar ejection forces should help identify the role of each mechanism.

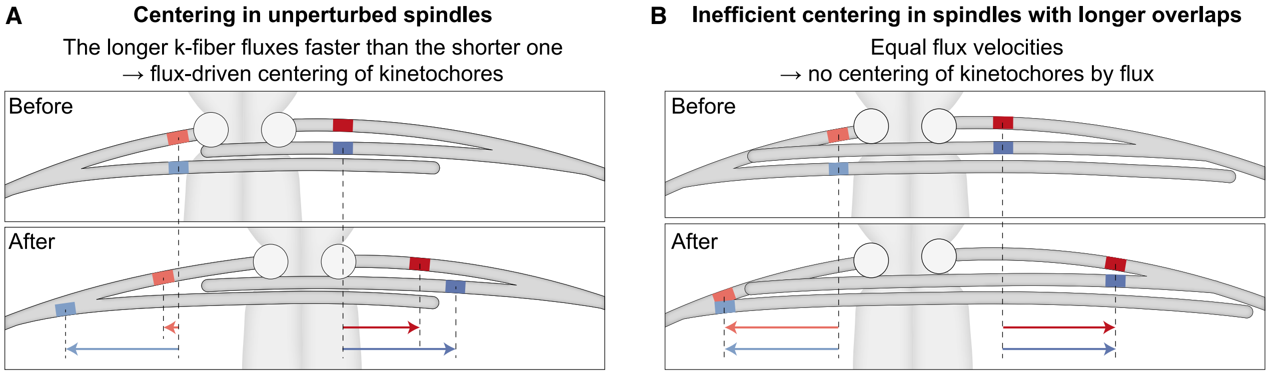

Figure 6. Mechanism by which poleward flux promotes kinetochore centering

(A) A pair of kinetochores (circles) is displaced toward the left (top). To visualize relative movements of the MTs, four marks are shown (red and blue). Over time (bottom), the marks on the bMTs move poleward by a similar distance (arrows), whereas the marks on the k-fibers move more slowly because of imperfect coupling between the bridging and k-fibers. Importantly, the longer k-fiber on the right side has a longer overlap with the bridging fiber and thus the coupling is stronger, leading to a higher flux velocity of this fiber in comparison with the shorter k-fiber, which in turn results in the movement of the kinetochores toward the spindle center.

(B) If the coupling between the k-fibers and the bridging fiber is too strong, such as in cases when the antiparallel overlaps are excessively long, the k-fibers flux velocity becomes similar to the velocity of the bridging fiber. Thus, k-fibers do not slide with respect to the bridging fiber, resulting in chromosome misalignment.

By developing a speckle microscopy assay to distinguish kMTs and bMTs, our work demonstrated that bMTs undergo poleward flux, and this flux is faster than that of kMTs. In contrast to metaphase, k-fibers and bridging fibers slide together at a similar rate in early anaphase (Vukušić et al., 2017), suggesting that tension between kinetochores slows down the flux of k-fibers in metaphase. Interestingly, slower flux of kMTs than adjacent non-kMTs was observed in *Xenopus* egg extracts (Maddox et al., 2003; Yang et al., 2008) and cranefly spermatocytes (LaFountain et al., 2004) in metaphase, indicating that the relationship between the flux of these two sets of MTs is conserved across organisms whose spindles undergo flux.

Two flux velocities have been observed also in human U2OS cells, where the subset of MTs fluxing faster than kMTs was associated with the γ-tubulin ring complex (γTuRC) (Lecland and Lüders, 2014). γTuRC is recruited to MTs by the augmin complex to nucleate new MTs (Kamasaki et al., 2013; Uehara et al., 2009; David et al., 2019; Goshima et al., 2008), including nucleation of bMTs along k-fibers (Manenica et al., 2020; O'Toole et al., 2020). Thus, the fast flux of bMTs in comparison with k-fibers measured here likely corresponds to the fast γTuRC-nucleated MT fraction. Our observation that the flux of bMTs slowed down after depletion of the augmin subunit Haus8 supports this conclusion.

The forces driving poleward flux have been debated, where the dominant forces are thought to be either at the spindle pole (Rogers et al., 2004; Ganem et al., 2005) or within interpolar MTs (Miyamoto et al., 2004; Brust-Mascher et al., 2009; Matos et al., 2009). Our experiments, which show that bridging fiber flux is largely unaffected by k-fibers, are in agreement with the latter possibility and support the assumption of our model that the leading forces are generated within antiparallel overlaps.

Our experiments together with the model suggests that NuMA transmits the force from the bMTs onto the kMTs. Interestingly, NuMA depletion was shown to cause asynchrony of MT poleward flux (Steblyanko et al., 2020), implying that NuMA crosslinks neighboring k-fibers and synchronizes their flux. We suggest that the synchrony in poleward flux of neighboring k-fibers is reflected in the correlated movement of neighboring kinetochore pairs (Vladimirou et al., 2013). In addition to NuMA, bMTs may promote synchrony in k-fiber flux as bMTs were shown to fan out at their ends and interact with neighboring k-fibers (O'Toole et al., 2020).

Depletion of PRC1 did not change the flux velocity of bMTs or kMTs, in agreement with a previous study (Steblyanko et al., 2020). Similarly, PRC1 depletion does not affect sliding of bMTs and spindle elongation in anaphase (Vukušić et al., 2021). As PRC1 depletion leads to ~50% decrease of the number of MTs in the bridging fiber in metaphase (Jagrić et al., 2021), our result suggests that the remaining bMTs are sufficient to generate flux. In contrast to PRC1, depletion of augmin, which decreases the number of bMTs to a larger extent than PRC1 (Manenica et al., 2020), led to slower bridging fiber flux. This was accompanied by slower k-fiber flux in agreement with the model prediction for slower sliding velocity of bMTs. Beside augmin, MKLP1 depletion led to a decrease in k-fiber and bridging fiber flux velocities. Given that MKLP1 localizes to the bridging fibers in metaphase and anaphase and is involved in anaphase spindle elongation (Jagrić et al., 2021; Vukušić et al., 2021), this motor may contribute to antiparallel sliding within bridging fiber overlaps. Additionally, CENP-E siRNA depletion reduced poleward flux rates, likely because of the role of CENP-E in targeting CLASPs, which promote flux, to kinetochores (Maiato et al., 2005; Maffini et al., 2009; Girão et al., 2020).

The physiological importance of chromosome alignment is in preventing lagging chromosomes and appearance of micronuclei, thereby promoting proper nuclear reformation and karyotype stability (Fonseca et al., 2019; Maiato et al., 2017). It will be interesting to explore the robustness of the flux-driven chromosome alignment and the resulting segregation fidelity in

healthy cells. Even more important, future work should reveal what aberrations in this mechanism lead to errors in chromosome segregation in cells with unstable karyotypes in which misaligned chromosomes appear.

Limitations of the study

Whereas in our model the relationship between k-fiber flux and kinetochore centering was observed by varying a single parameter, experimental perturbations relied on depletion of motor proteins, which have multiple functions within the spindle. Thus, the interpretation in light of the model is not always straightforward. For example, depletion of Kif18A led to misaligned kinetochores, longer overlaps of bMTs, and faster kMT flux. However, it is not clear to what extent kinetochore misalignment is due to perturbed flux-driven centering or perturbed regulation of k-fiber length at the plus end in the absence of Kif18A (Stumpff et al., 2012; Du et al., 2010).

In contrast to kinetochore misalignment observed after depletions of Kif18A alone or in combination with Kif4A and PRC1, depletion of Kif4A alone showed no effect on kinetochore alignment. Yet kinetochore misalignment was expected because the overlaps of bMTs were extended and the k-fiber flux was faster. Proper kinetochore alignment in the absence of Kif4A is possibly due to the activity of Kif18A at the k-fiber tips.

STAR★METHODS

Detailed methods are provided in the online version of this paper and include the following:

- KEY RESOURCES TABLE
- RESOURCE AVAILABILITY
  - Lead contact
  - Materials and availability
  - Data and code availability
- EXPERIMENTAL MODEL AND SUBJECT DETAILS
- METHOD DETAILS
  - Theory for kinetochore centering
  - Solution of the model
  - Choice of parameters
  - Cell culture
  - RNA interference and transfection
  - Speckle microscopy
  - Immunostaining
  - Photoactivation assay
- QUANTIFICATION AND STATISTICAL ANALYSIS
  - Image analysis

SUPPLEMENTAL INFORMATION

Supplemental information can be found online at https://doi.org/10.1016/j.celrep.2022.111169.

ACKNOWLEDGMENTS

We thank Alexey Khodjakov, Marin Barišić, Helder Maiato, and Andrew McAinsh for the cell lines; Ivana Šarić for the drawings; and all members of Tolić and Pavin groups for helpful discussions. This work was funded by the European Research Council (ERC Synergy Grant, GA number 855158, to I.M.T. and N.P., and ERC Consolidator Grant, GA number 647077, to I.M.T.), the Croatian Science Foundation (HRZZ; grants PZS-2019-02-7653 to I.M.T. and IP-2019-04-5967 to N.P.), and Croatian Government and European Union through the European Regional Development Fund—the Competitiveness and Cohesion Operational Programme: IPSted (grant KK.01.1.1.04.0057) and QuantiXLie Center of Excellence (grant KK.01.1.1.01.0004). The work of doctoral students M.J. and A.B. was supported by the "Young Researchers' Career Development Project – Training of Doctoral Students" of the Croatian Science Foundation.

AUTHOR CONTRIBUTIONS

P.R. and M.J. performed all experiments. P.R. analyzed the data with help of M.J. D.B. developed the model, on the basis of A.B.'s pilot work. N.P. and I.M.T. conceived the project and supervised the theory and experiments.

DECLARATION OF INTERESTS

The authors declare no competing interests.

Received: September 15, 2021
Revised: April 20, 2022
Accepted: July 14, 2022
Published: August 2, 2022

REFERENCES

Akiyoshi, B., Sarangapani, K.K., Powers, A.F., Nelson, C.R., Reichow, S.L., Arellano-Santoyo, H., Gonen, T., Ranish, J.A., Asbury, C.L., and Biggins, S. (2010). Tension directly stabilizes reconstituted kinetochore-microtubule attachments. Nature *468*, 576–579. https://doi.org/10.1038/nature09594.

Armond, J.W., Harry, E.F., McAinsh, A.D., and Burroughs, N.J. (2015). Inferring the forces controlling metaphase kinetochore oscillations by reverse engineering system dynamics. PLoS Comput. Biol. *11*, e1004607. https://doi.org/10.1371/journal.pcbi.1004607.

Brust-Mascher, I., Sommi, P., Cheerambathur, D.K., and Scholey, J.M. (2009). Kinesin-5-dependent poleward flux and spindle length control in Drosophila embryo mitosis. Mol. Biol. Cell *20*, 1749–1762. https://doi.org/10.1091/mbc.e08-10-1033.

Buđa, R., Vukušić, K., and Tolić, I.M. (2017). Dissection and characterization of microtubule bundles in the mitotic spindle using femtosecond laser ablation. Methods Cell Biol. *139*, 81–101. https://doi.org/10.1016/bs.mcb.2016.11.007.

Cheeseman, I.M., Chappie, J.S., Wilson-Kubalek, E.M., and Desai, A. (2006). The conserved KMN network constitutes the core microtubule-binding site of the kinetochore. Cell *127*, 983–997. https://doi.org/10.1016/j.cell.2006.09.039.

Cheeseman, I.M., and Desai, A. (2008). Molecular architecture of the kinetochore-microtubule interface. Nat. Rev. Mol. Cell Biol. *9*, 33–46. https://doi.org/10.1038/nrm2310.

Civelekoglu-Scholey, G., He, B., Shen, M., Wan, X., Roscioli, E., Bowden, B., and Cimini, D. (2013). Dynamic bonds and polar ejection force distribution explain kinetochore oscillations in PtK1 cells. J. Cell Biol. *201*, 577–593. https://doi.org/10.1083/jcb.201301022.

Civelekoglu-Scholey, G., Sharp, D.J., Mogilner, A., and Scholey, J.M. (2006). Model of chromosome motility in Drosophila embryos: adaptation of a general mechanism for rapid mitosis. Biophys. J. *90*, 3966–3982. https://doi.org/10.1529/biophysj.105.078691.

David, A.F., Roudot, P., Legant, W.R., Betzig, E., Danuser, G., and Gerlich, D.W. (2019). Augmin Accumulation on long-lived microtubules drives amplification and kinetochore-directed growth. J. Cell Biol. *218*, 2150–2168. https://doi.org/10.1083/jcb.201805044.

Du, Y., English, C.A., and Ohi, R. (2010). The kinesin-8 Kif18A dampens microtubule plus-end dynamics. Curr. Biol. *20*, 374–380. https://doi.org/10.1016/j.cub.2009.12.049.

CellPress
OPEN ACCESS

Dudka, D., Noatynska, A., Smith, C.A., Liaudet, N., McAinsh, A.D., and Meraldi, P. (2018). Complete microtubule-kinetochore occupancy favours the segregation of merotelic attachments. Nat. Commun. *9*, 2042. https://doi.org/10.1038/s41467-018-04427-x.

Elting, M.W., Prakash, M., Udy, D.B., and Dumont, S. (2017). Mapping load-bearing in the mammalian spindle reveals local kinetochore fiber anchorage that provides mechanical isolation and redundancy. Curr. Biol. *27*, 2112–2122.e5. https://doi.org/10.1016/j.cub.2017.06.018.

Fonseca, C.L., Malaby, H.L.H., Sepaniac, L.A., Martin, W., Byers, C., Czechanski, A., Messinger, D., Tang, M., Ohi, R., Reinholdt, L.G., and Stumpff, J. (2019). Mitotic chromosome alignment ensures mitotic fidelity by promoting interchromosomal compaction during anaphase. J. Cell Biol. *218*, 1148–1163. https://doi.org/10.1083/jcb.201807228.

Forer, A. (1965). Local reduction of spindle fiber birefringence in living Nephrotoma suturalis (Loew) spermatocytes induced by ultraviolet microbeam irradiation. J. Cell Biol. *25*, 95–117. https://doi.org/10.1083/jcb.25.1.95.

Ganem, N.J., Upton, K., and Compton, D.A. (2005). Efficient mitosis in human cells lacking poleward microtubule flux. Curr. Biol. *15*, 1827–1832. https://doi.org/10.1016/j.cub.2005.08.065.

Gardner, M.K., Bouck, D.C., Paliulis, L.V., Meehl, J.B., O'Toole, E.T., Haase, J., Soubry, A., Joglekar, A.P., Winey, M., Salmon, E.D., et al. (2008). Chromosome congression by Kinesin-5 motor-mediated disassembly of longer kinetochore microtubules. Cell *135*, 894–906. https://doi.org/10.1016/j.cell.2008.09.046.

Gergely, Z.R., Crapo, A., Hough, L.E., McIntosh, J.R., and Betterton, M.D. (2016). Kinesin-8 effects on mitotic microtubule dynamics contribute to spindle function in fission yeast. Mol. Biol. Cell *27*, 3490–3514. https://doi.org/10.1091/mbc.E15-07-0505.

Girão, H., Okada, N., Rodrigues, T.A., Silva, A.O., Figueiredo, A.C., Garcia, Z., Moutinho-Santos, T., Hayashi, I., Azevedo, J.E., Macedo-Ribeiro, S., and Maiato, H. (2020). CLASP2 binding to curved microtubule tips promotes flux and stabilizes kinetochore attachments. J. Cell Biol. *219*, e201905080. https://doi.org/10.1083/jcb.201905080.

Goshima, G., Mayer, M., Zhang, N., Stuurman, N., and Vale, R.D. (2008). Augmin: A protein complex required for centrosome-independent microtubule generation within the spindle. J. Cell Biol. *181*, 421–429. https://doi.org/10.1083/jcb.200711053.

Hamaguchi, Y., Toriyama, M., Sakai, H., and Hiramoto, Y. (1987). Redistribution of fluorescently labeled tubulin in the mitotic apparatus of sand dollar eggs and the effects of taxol. Cell Struct. Funct. *12*, 43–52. https://doi.org/10.1247/csf.12.43.

Hentrich, C., and Surrey, T. (2010). Microtubule organization by the antagonistic mitotic motors kinesin-5 and kinesin-14. J. Cell Biol. *189*, 465–480. https://doi.org/10.1083/jcb.200910125.

Hiramoto, Y., and Izutsu, K. (1977). Poleward movement of "markers" existing in mitotic spindles of grasshopper spermatocytes. Cell Struct. Funct. *2*, 257–259. https://doi.org/10.1247/csf.2.257.

Jagrić, M., Risteski, P., Martinčić, J., Milas, A., and Tolić, I.M. (2021). Optogenetic control of PRC1 reveals its role in chromosome alignment on the spindle by overlap length-dependent forces. Elife *10*, e61170. https://doi.org/10.7554/eLife.61170.

Joglekar, A.P., and Hunt, A.J. (2002). A simple, mechanistic model for directional instability during mitotic chromosome movements. Biophys. J. *83*, 42–58. https://doi.org/10.1016/S0006-3495(02)75148-5.

Kajtez, J., Solomatina, A., Novak, M., Polak, B., Vukušić, K., Rüdiger, J., Cojoc, G., Milas, A., Šumanovac Šestak, I., Risteski, P., et al. (2016). Overlap microtubules link sister k-fibres and balance the forces on bi-oriented kinetochores. Nat. Commun. *7*, 10298. https://doi.org/10.1038/ncomms10298.

Kamasaki, T., O'Toole, E., Kita, S., Osumi, M., Usukura, J., McIntosh, J.R., and Goshima, G. (2013). Augmin-dependent microtubule nucleation at microtubule walls in the spindle. J. Cell Biol. *202*, 25–33. https://doi.org/10.1083/jcb.201304031.

Ke, K., Cheng, J., and Hunt, A.J. (2009). The distribution of polar ejection forces determines the amplitude of chromosome directional instability. Curr. Biol. *19*, 807–815. https://doi.org/10.1016/j.cub.2009.04.036.

Klemm, A.H., Bosilj, A., Glunčić, M., Pavin, N., and Tolić, I.M. (2018). Metaphase kinetochore movements are regulated by kinesin-8 motors and microtubule dynamic instability. Mol. Biol. Cell *29*, 1332–1345. https://doi.org/10.1091/mbc.E17-11-0667.

LaFountain, J.R., Jr., Cohan, C.S., Siegel, A.J., and LaFountain, D.J. (2004). Direct visualization of microtubule flux during metaphase and anaphase in crane-fly spermatocytes. Mol. Biol. Cell *15*, 5724–5732. https://doi.org/10.1091/mbc.e04-08-0750.

Lecland, N., and Lüders, J. (2014). The dynamics of microtubule minus ends in the human mitotic spindle. Nat. Cell Biol. *16*, 770–778. https://doi.org/10.1038/ncb2996.

Lukinavičius, G., Reymond, L., D'Este, E., Masharina, A., Göttfert, F., Ta, H., Güther, A., Fournier, M., Rizzo, S., Waldmann, H., et al. (2014). Fluorogenic probes for live-cell imaging of the cytoskeleton. Nat. Methods *11*, 731–733. https://doi.org/10.1038/nmeth.2972.

Maddox, P., Straight, A., Coughlin, P., Mitchison, T.J., and Salmon, E.D. (2003). Direct observation of microtubule dynamics at kinetochores in Xenopus extract spindles: implications for spindle mechanics. J. Cell Biol. *162*, 377–382. https://doi.org/10.1083/jcb.200301088.

Maffini, S., Maia, A.R.R., Manning, A.L., Maliga, Z., Pereira, A.L., Junqueira, M., Shevchenko, A., Hyman, A., Yates, J.R., 3rd, Galjart, N., et al. (2009). Motor-independent targeting of CLASPs to kinetochores by CENP-E promotes microtubule turnover and poleward flux. Curr. Biol. *19*, 1566–1572. https://doi.org/10.1016/j.cub.2009.07.059.

Maiato, H., Gomes, A.M., Sousa, F., and Barisic, M. (2017). Mechanisms of chromosome congression during mitosis. Biology *6*, 13. https://doi.org/10.3390/biology6010013.

Maiato, H., Khodjakov, A., and Rieder, C.L. (2005). Drosophila CLASP is required for the incorporation of microtubule subunits into fluxing kinetochore fibres. Nat. Cell Biol. *7*, 42–47. https://doi.org/10.1038/ncb1207.

Manenica, M., Štimac, V., Koprivec, I., Simunić, J., and Tolić, I.M. (2020). Augmin regulates kinetochore tension and spatial arrangement of spindle microtubules by nucleating bridging fibers. Preprint at bioRxiv. https://doi.org/10.1101/2020.09.10.291740.

Mary, H., Fouchard, J., Gay, G., Reyes, C., Gauthier, T., Gruget, C., Pécréaux, J., Tournier, S., and Gachet, Y. (2015). Fission yeast kinesin-8 controls chromosome congression independently of oscillations. J. Cell Sci. *128*, 3720–3730. https://doi.org/10.1242/jcs.160465.

Matos, I., Pereira, A.J., Lince-Faria, M., Cameron, L.A., Salmon, E.D., and Maiato, H. (2009). Synchronizing chromosome segregation by flux-dependent force equalization at kinetochores. J. Cell Biol. *186*, 11–26. https://doi.org/10.1083/jcb.200904153.

Mayr, M.I., Hümmer, S., Bormann, J., Grüner, T., Adio, S., Woehlke, G., and Mayer, T.U. (2007). The human kinesin Kif18A is a motile microtubule depolymerase essential for chromosome congression. Curr. Biol. *17*, 488–498. https://doi.org/10.1016/j.cub.2007.02.036.

Mitchison, T.J. (1989). Polewards microtubule flux in the mitotic spindle: evidence from photoactivation of fluorescence. J. Cell Biol. *109*, 637–652. https://doi.org/10.1083/jcb.109.2.637.

Mitchison, T.J. (2005). Mechanism and function of poleward flux in Xenopus extract meiotic spindles. Philos. Trans. R. Soc. Lond. B Biol. Sci. *360*, 623–629. https://doi.org/10.1098/rstb.2004.1616.

Miyamoto, D.T., Perlman, Z.E., Burbank, K.S., Groen, A.C., and Mitchison, T.J. (2004). The kinesin Eg5 drives poleward microtubule flux in Xenopus laevis egg extract spindles. J. Cell Biol. *167*, 813–818. https://doi.org/10.1083/jcb.200407126.

O'Toole, E., Morphew, M., and McIntosh, J.R. (2020). Electron tomography reveals aspects of spindle structure important for mechanical stability at metaphase. Mol. Biol. Cell *31*, 184–195. https://doi.org/10.1091/mbc.E19-07-0405.

Ohi, R., Coughlin, M.L., Lane, W.S., and Mitchison, T.J. (2003). An inner centromere protein that stimulates the microtubule depolymerizing activity of a KinI kinesin. Dev. Cell *5*, 309–321. https://doi.org/10.1016/s1534-5807(03)00229-6.

Pavin, N., and Tolić, I.M. (2021). Mechanobiology of the mitotic spindle. Dev. Cell *56*, 192–201. https://doi.org/10.1016/j.devcel.2020.11.003.

Polak, B., Risteski, P., Lesjak, S., and Tolić, I.M. (2017). PRC1-labeled microtubule bundles and kinetochore pairs show one-to-one association in metaphase. EMBO Rep. *18*, 217–230. https://doi.org/10.15252/embr.201642650.

Rieder, C.L., and Salmon, E.D. (1994). Motile kinetochores and polar ejection forces dictate chromosome position on the vertebrate mitotic spindle. J. Cell Biol. *124*, 223–233. https://doi.org/10.1083/jcb.124.3.223.

Rogers, G.C., Rogers, S.L., Schwimmer, T.A., Ems-McClung, S.C., Walczak, C.E., Vale, R.D., Scholey, J.M., and Sharp, D.J. (2004). Two mitotic kinesins cooperate to drive sister chromatid separation during anaphase. Nature *427*, 364–370. https://doi.org/10.1038/nature02256.

Shimamoto, Y., Forth, S., and Kapoor, T.M. (2015). Measuring pushing and braking forces generated by ensembles of kinesin-5 crosslinking two microtubules. Dev. Cell *34*, 669–681. https://doi.org/10.1016/j.devcel.2015.08.017.

Shrestha, R.L., Conti, D., Tamura, N., Braun, D., Ramalingam, R.A., Cieslinski, K., Ries, J., and Draviam, V.M. (2017). Aurora-B kinase pathway controls the lateral to end-on conversion of kinetochore-microtubule attachments in human cells. Nat. Commun. *8*, 150. https://doi.org/10.1038/s41467-017-00209-z.

Steblyanko, Y., Rajendraprasad, G., Osswald, M., Eibes, S., Jacome, A., Geley, S., Pereira, A.J., Maiato, H., and Barisic, M. (2020). Microtubule poleward flux in human cells is driven by the coordinated action of four kinesins. EMBO J. *39*, e105432. https://doi.org/10.15252/embj.2020105432.

Stumpff, J., von Dassow, G., Wagenbach, M., Asbury, C., and Wordeman, L. (2008). The kinesin-8 motor Kif18A suppresses kinetochore movements to control mitotic chromosome alignment. Dev. Cell *14*, 252–262. https://doi.org/10.1016/j.devcel.2007.11.014.

Stumpff, J., Wagenbach, M., Franck, A., Asbury, C.L., and Wordeman, L. (2012). Kif18A and chromokinesins confine centromere movements via microtubule growth suppression and spatial control of kinetochore tension. Dev. Cell *22*, 1017–1029. https://doi.org/10.1016/j.devcel.2012.02.013.

Tolić, I.M., and Pavin, N. (2021). Mitotic spindle: lessons from theoretical modeling. Mol. Biol. Cell *32*, 218–222. https://doi.org/10.1091/mbc.E20-05-0335.

Uehara, R., Nozawa, R.s., Tomioka, A., Petry, S., Vale, R.D., Obuse, C., and Goshima, G. (2009). The augmin complex plays a critical role in spindle microtubule generation for mitotic progression and cytokinesis in human cells. Proc. Natl. Acad. Sci. USA *106*, 6998–7003. https://doi.org/10.1073/pnas.0901587106.

Valentine, M.T., Fordyce, P.M., Krzysiak, T.C., Gilbert, S.P., and Block, S.M. (2006). Individual dimers of the mitotic kinesin motor Eg5 step processively and support substantial loads in vitro. Nat. Cell Biol. *8*, 470–476. https://doi.org/10.1038/ncb1394.

Varga, V., Helenius, J., Tanaka, K., Hyman, A.A., Tanaka, T.U., and Howard, J. (2006). Yeast kinesin-8 depolymerizes microtubules in a length-dependent manner. Nat. Cell Biol. *8*, 957–962. https://doi.org/10.1038/ncb1462.

Vladimirou, E., Mchedlishvili, N., Gasic, I., Armond, J.W., Samora, C.P., Meraldi, P., and McAinsh, A.D. (2013). Nonautonomous movement of chromosomes in mitosis. Dev. Cell *27*, 60–71. https://doi.org/10.1016/j.devcel.2013.08.004.

Vukušić, K., Buđa, R., Bosilj, A., Milas, A., Pavin, N., and Tolić, I.M. (2017). Microtubule sliding within the bridging fiber pushes kinetochore fibers apart to segregate chromosomes. Dev. Cell *43*, 11–23.e6. https://doi.org/10.1016/j.devcel.2017.09.010.

Vukušić, K., Ponjavić, I., Buđa, R., Risteski, P., and Tolić, I.M. (2021). Microtubule-sliding modules based on kinesins EG5 and PRC1-dependent KIF4A drive human spindle elongation. Dev. Cell *56*, 1253–1267.e10. https://doi.org/10.1016/j.devcel.2021.04.005.

Waterman-Storer, C.M., Desai, A., Bulinski, J.C., and Salmon, E.D. (1998). Fluorescent speckle microscopy, a method to visualize the dynamics of protein assemblies in living cells. Curr. Biol. *8*, 1227–1230. https://doi.org/10.1016/s0960-9822(07)00515-5.

Waterman-Storer, C.M., and Salmon, E.D. (1998). How microtubules get fluorescent speckles. Biophys. J. *75*, 2059–2069. https://doi.org/10.1016/S0006-3495(98)77648-9.

West, R.R., Malmstrom, T., and McIntosh, J.R. (2002). Kinesins klp5+ and klp6+ are required for normal chromosome movement in mitosis. J. Cell Sci. *115*, 931–940. https://doi.org/10.1242/jcs.115.5.931.

Yang, G., Cameron, L.A., Maddox, P.S., Salmon, E.D., and Danuser, G. (2008). Regional variation of microtubule flux reveals microtubule organization in the metaphase meiotic spindle. J. Cell Biol. *182*, 631–639. https://doi.org/10.1083/jcb.200801105.

STAR★METHODS

KEY RESOURCES TABLE

REAGENT or RESOURCE	SOURCE	IDENTIFIER
**Antibodies**		
Rabbit polyclonal anti-Kif18A	Bethyl Laboratories	Cat# A301-080A; RRID: AB_2296551
Mouse monoclonal anti-Kif4A (E-8)	Santa Cruz Biotechnology	Cat# sc-365144; RRID: AB_10707683
Mouse monoclonal anti-Kid (B-9)	Santa Cruz Biotechnology	Cat# sc-390640
Rabbit polyclonal anti-CENP-E	Sigma-Aldrich	Cat# C7488; RRID: AB_476868
Rabbit monoclonal anti-MKLP1 [EPR10879]	Abcam	Cat# ab174304
Mouse monoclonal anti-PRC1 (C-1)	Santa Cruz Biotechnology	Cat# sc-376983
Rabbit polyclonal anti-Haus8/HICE1	Invitrogen	Cat# PA5-21331; RRID: AB_11153508
Mouse monoclonal anti-NuMA (F-11)	Santa Cruz Biotechnology	Cat# sc-365532; RRID: AB_10846197
Mouse monoclonal anti-Astrin (C-1)	Sigma-Aldrich	Cat# MABN2487
Donkey anti-mouse IgG Alexa Fluor 594 preadsorbed	Abcam	Cat# ab150112; RRID: AB_2813898
Donkey anti-rabbit IgG Alexa Fluor 594 preadsorbed	Abcam	Cat# ab150064; RRID: AB_2734146
Donkey Anti-Rabbit IgG Alexa Fluor 647	Abcam	Cat# ab150075; RRID: AB_2752244
**Chemicals, peptides, and recombinant proteins**		
Dulbecco's Modified Eagle Medium	Capricorn Scientific	Cat# DMEM-HPSTA
Fetal Bovine Serum	Sigma-Aldrich	Cat# F2442
Penicillin/Streptomycin	Lonza	Cat# DE17-502E
Opti-MEM Reduced Serum Medium	Gibco	Cat# 31985047
Normal Goat Serum	Invitrogen	Cat# 31872
Phosphate Buffered Saline	Roth	Cat# 9143.1
Methanol	Sigma-Aldrich	Cat# 32213-M
Formaldehyde	Biognost	Cat# FNB4
Triton-X-100	Sigma-Aldrich	Cat# 93426
ZM447439	Selleckchem	Cat# S1103
MG-132	Sigma-Aldrich	Cat# 474790
**Critical commercial assays**		
Lipofectamine RNAiMAX Transfection Reagent	Invitrogen	Cat# 13778150
SiR-Tubulin	Spirochrome AG	Cat# SC002
Mycoalert Mycoplasma Detection Kit	Lonza	Cat# LT07-218
**Experimental models: Cell lines**		
hTERT-RPE-1 cell line (human retinal pigmented epithelium, female) permanently transfected and stabilized using CENP-A-GFP and centrin1-GFP	Laboratory of Alexey Khodjakov, Wadsworth Center, New York State Department of Health, Albany, NY	N/A
U2OS cell lines (human osteosarcoma, female), permanently transfected and stabilized using CENP-A-GFP, and photoactivatable PA-GFP-α-tubulin, CENP-A-GFP and mCherry-α-tubulin	Laboratory of Helder Maiato, Institute for Molecular Cell Biology, University of Porto, Portugal; Laboratory of Marin Barišić, Danish Cancer Society Research Center, Copenhagen, Denmark	N/A
HeLa cell line (human cervical adenocarcinoma, female) permanently transfected with EGFP–CENP-A	Laboratory of Andrew McAinsh, Centre for Mechanochemical Cell Biology, University of Warwick, Coventry, UK	N/A

(Continued on next page)

Continued		
REAGENT or RESOURCE	SOURCE	IDENTIFIER
Oligonucleotides		
Human Kif18A siRNA	Ambion	#Cat 4390825, ID: s37882
Human Kif4A siRNA	Santa Cruz Biotechnology	#Cat sc-60888
Human Kif22/Kid siRNA	Ambion	#Cat 4392420, ID: s7911
Human CENP-E siRNA	Dharmacon	#Cat L-003252-000010
Human MKLP1 siRNA	Santa Cruz Biotechnology	#Cat sc-35936
Human PRC1 siRNA	Dharmacon	#Cat L-019491-00-0010
Human Haus8 siRNA	Dharmacon	#Cat L-031247-01-0005
Human NuMA siRNA	Santa Cruz Biotechnology	#Cat sc-43978
Human Ndc80 siRNA	Merck	#Cat HA12977117-004
Software and algorithms		
ImageJ	National Institutes of Health	https://imagej.nih.gov/ij/
R Studio	RStudio	https://www.rstudio.com
MATLAB	The MathWorks	https://uk.mathworks.com
SciDavis	Free Software Foundation	http://scidavis.sourceforge.net
Adobe Illustrator CC	Adobe Systems	https://www.adobe.com

RESOURCE AVAILABILITY

Lead contact

Further information and requests for resources should be directed to and will be fulfilled by the lead contact, Iva M. Tolić (tolic@irb.hr).

Materials and availability

This study did not generate new unique reagents.

Data and code availability

All data reported in this paper will be shared by the lead contact upon request. This paper does not report original code. Any additional information required to reanalyze the data reported in this paper is available from the lead contact upon request.

EXPERIMENTAL MODEL AND SUBJECT DETAILS

hTERT-RPE-1 cell line (female) with a stable expression of CENP-A-GFP and centrin1-GFP was a gift from Alexey Khodjakov (Wadsworth Center, New York State Department of Health, Albany, NY, USA). U2OS cell lines (female) expressing CENP-A-GFP and photoactivatable PA-GFP-α-tubulin, CENP-A-GFP and mCherry-α-tubulin were a gift from Marin Barišić (Danish Cancer Society Research Center, Copenhagen, Denmark) and Helder Maiato (Institute for Molecular Cell Biology, University of Porto, Portugal). HeLa cell line (female) with a stable expression of EGFP-CENP-A was a gift from Andrew McAinsh (Centre for Mechanochemical Cell Biology, University of Warwick, Coventry, UK).

METHOD DETAILS

Theory for kinetochore centering

The model describes a system consisting of two sister kinetochores, two MTs representing the left and right k-fiber, which extend from the spindle edges to the kinetochores, and two bridging MTs which extend from the edges and interdigitate in the middle (Figures 1A and S1D). The positions of the sister kinetochores are denoted by $x^{\pm}_{KC}$, while the positions of k-fibers and bridging fibers are taken as arbitrary positions along their lattice and are denoted by $x^{\pm}_{kMT}$ and $x^{\pm}_{bMT}$, respectively. All these positions change in time t and their velocities are calculated as $v^{\pm}_i = dx^{\pm}_i/dt$, for i = KC, kMT, bMT. Hereon the superscripts + and − denote the right and left sides of the model, respectively. Note that the growth velocity of k-fibers is not explicitly described, but can be calculated as $v^{\pm}_{KC} - v^{\pm}_{kMT}$. The length of the MT overlap within the bridging fiber is denoted D_0 and the spindle length is denoted L_0.

In order to calculate the movement of the kinetochores we first describe forces at them. The elastic connection between sister kinetochores is described by a force exerted by Hookean spring, $F_{el} = k(x^{+}_{KC} - x^{-}_{KC} - x_0)$, where k denotes the elastic coefficient and x_0 the spring rest length. Kinetochores also interact with MTs and the force exerted by MT plus end is described by $F^{\pm}_{KC} = -\mu_{KC}(v^{\pm}_{KC} - v^{\pm}_{kMT})$. Here, μ_{KC} denotes the effective friction coefficient at the kinetochore. This description is a

simplification of the force-velocity relationship measured for kinetochores (Akiyoshi et al., 2010). Because these forces, exerted by the elastic connection and the MT, are the only forces acting at kinetochores in our model, they balance each other,

$$F_{KC}^{\pm} = \pm F_{el}. \quad \text{(Equation 4)}$$

Equation (4) also include the balance of forces between sister kinetochores, $F_{KC}^{+} = -F_{KC}^{-}$.

The movement of the k-fiber is driven by forces exerted by molecular motors distributed along the k-fiber and bridging MT overlap, $F_m^{\pm}$. These forces are opposed by the damping force of the cross-linking proteins, $F_c^{\pm}$, and by the force at the kinetochore,

$$F_m^{\pm} - F_c^{\pm} - F_{KC}^{\pm} = 0. \quad \text{(Equation 5)}$$

This expression is an application of Equation (3) to left and right k-fibers.

The forces of the motors distributed along the antiparallel overlap of a k-fiber and a bridging fiber are described by Equation (1), which for left and right sides reads $F_m^{\pm} = D^{\pm} n_m f_m^{\pm}$. Force exerted by a single motor depends on the relative velocity of the k-fiber and the bridging fiber of opposite orientation and is described through a linear force-velocity relation $v_{kMT}^{\pm} - v_{bMT}^{\mp} = v_0\left[\pm 1 - f_m^{\pm}/f_0\right]$, where f_0 denotes the stall force and v_0 the velocity without a load. The linear density of the motors is denoted n_m, and the length of the antiparallel overlap of the bridging and k-fiber is given by $D^{\pm} = (D_0/2 \mp x_{KC}^{\pm})\theta(D_0/2 \mp x_{KC}^{\pm})$, where θ is the Heaviside step function which ensures that the antiparallel overlap exists. The number of motors is given as $N_m^{\pm} = n_m D^{\pm}$.

The damping force of the crosslinking proteins is given by Equation (2), which for left and right sides reads $F_c^{\pm} = N_c^{\pm} f_c^{\pm}$, where the damping force of a single crosslinker, $f_c^{\pm} = \mu_c (v_{kMT}^{\pm} - v_{bMT}^{\pm})$, depends on the friction coefficient of a crosslinking protein, μ_c, and the relative velocity of the k-fiber and the bridging fiber. The number of passive crosslinkers distributed along the parallel overlap of a k-fiber and a bridging fiber, $N_c^{\pm} = n_c L^{\pm}$, is calculated from linear density n_c and the length of the k-fiber $L^{\pm} = \pm(L_0/2 - x_{KC}^{\pm})$.

The movement of the bMTs is driven by the force exerted by motors distributed along the antiparallel overlap of bMTs, F_{bMT}. This force is opposed by the motor forces exerted along the antiparallel overlap of the bridging fiber and the k-fiber and by the damping force of the crosslinking proteins exerted along the parallel overlap of the bridging fiber and the k-fiber,

$$F_{bMT} \pm F_m^{\mp} \pm F_c^{\pm} = 0. \quad \text{(Equation 6)}$$

The force exerted in the overlap of bMTs depends on their relative velocities, $F_{bMT} = N_{bMT} f_0 \left[1 - (v_{bMT}^{+} - v_{bMT}^{-})/v_0\right]$, and the number of motors in the overlap of bMTs, which is given as $N_{bMT} = n_m D_0$.

Solution of the model

Approximations in the model

Even though the model can be solved as given, we introduce two approximations that apply, to a large extent, to the studied spindles. First, we neglect the difference in kinetochore velocities, $\Delta v_{KC} \equiv v_{KC}^{+} - v_{KC}^{-} = 0$, based on the following arguments. Interkinetochore velocity on a time scale relevant for centering of kinetochores $t_c = D_0/(v_{KC}^{+} + v_{KC}^{-})$ has an approximate value $\Delta v_{KC} \approx (x_{KC}^{+} - x_{KC}^{-})/t_c$. By applying Equation (4) on the left and right sides, the normalized interkinetochore velocity reads

$$\frac{\Delta v_{KC}}{v_{KC}^{+} + v_{KC}^{-}} = \left[\frac{2kD_0}{\mu_{KC} v_0} + \frac{v_{KC}^{+} + v_{KC}^{-}}{v_0}\right]^{-1}, \quad \text{(Equation 7)}$$

where we use $v_0 = v_{kMT}^{+} - v_{kMT}^{-}$ as an upper limit for k-fiber velocity difference. In the case of parameters that are relevant for our system, spring constant representing elasticity of the chromosomes, k = 100 pN/μm (Joglekar and Hunt, 2002), and D_0, μ_{KC}, and v_0 from Figure 1B, the first term of the right-hand side of Equation (7) obeys $2kD_0/\mu_{KC} v_0 \gg 1$. In this limit the right-hand side of Equation (7) approaches zero and the interkinetochore velocity can be neglected.

Second, we set the velocities of the bMTs to the value $v_{bMT}^{\pm} = \pm v_0/2$, based on the following approximation. For kinetochores in the central position, the overlap regions on the left and right sides have the same length, $D^{+} = D^{-}$ and $L^{+} = L^{-}$. By applying this symmetry to Equation (6) we derive an expression:

$$\frac{2v_{bMT}^{+}}{v_0}\left(1 + \frac{N_c^{+}\mu_c}{N_{bMT} f_0}\frac{v_0}{2} + \frac{D^{+}}{2D_0}\right) = 1 + \frac{N_c^{+}\mu_c}{N_{bMT} f_0} v_{kMT}^{+} - \frac{D^{+}}{D_0}\left(-1 - \frac{v_{kMT}^{-}}{v_0}\right). \quad \text{(Equation 8)}$$

In the case where the contribution of the motors dominates over that of cross-linkers, $N_c^{+}\mu_c v_0 \ll 2N_{bMT} f_0$, the second term in the bracket on the left side of the equation is much smaller than 1. Analogously, the second term on the right side can be neglected. Because the length of the antiparallel overlap between bridging and k-fibers is much smaller than the length of the overlap between bridging fibers, $D^{+} \ll D_0$, the third terms on both sides of the equation can be neglected. In this limit, Equation (8) reduces to $2v_{bMT}^{+}/v_0 = 1$. Additionally, we calculated the bMT flux for the parameters as in Figure 1C from the force balance of the entire system, given by Equations 4, 5 and 6, and found that the relative deviations of the calculated bMT flux from the value $v_0/2$ changed during kinetochore centering from 5.5% to 12%, whereas for a longer bridging MT overlap, D_0= 8 μm, the deviations were 7.5% to 10%.

Velocities of k-fibers and kinetochores

By applying these approximations, force-velocity relationship for individual motors, friction forces exerted by passive crosslinkers and kinetochores to Equation (5) we derive expressions for k-fiber velocities:

$$v_{kMT}^{\pm} = \alpha^{\pm}\left(\mu_{KC}v_{KC} \pm \frac{v_0}{2}\left(g_c^{\pm} + g_m^{\pm}\right)\right). \quad \text{(Equation 9)}$$

Here, to have shorter notations in our model, we define three symbols: $\alpha^{\pm} \equiv \left(N_c^{\pm}\mu_c + N_m^{\pm}f_0/v_0 + \mu_{KC}\right)^{-1}$, $g_c^{\pm} \equiv N_c^{\pm}\mu_c$, and $g_m^{\pm} \equiv N_m^{\pm}f_0/v_0$.

Next, by combining the force balance on sister kinetochores, obtained from Equation (4), with Equation (5), we obtain a balance of forces between left and right k-fibers, which are exerted by motors and crosslinkers, $F_c^{+} - F_m^{+} = -F_c{}^{-} + F_m{}^{-}$. By applying force-velocity relationship for individual motors and friction forces exerted by passive crosslinkers to this expression, in combination with Equation (9), we obtain a final expression for kinetochore velocities:

$$v_{KC} = \frac{v_0}{2\mu_{KC}} \frac{\left(g_c^{+} + g_m^{+}\right)\left(1 - \alpha^{+}\left(g_c^{+} + g_m^{+}\right)\right) - \left(g_c^{-} + g_m^{-}\right)\left(1 - \alpha^{-}\left(g_c^{-} + g_m^{-}\right)\right)}{\alpha^{+}\left(g_c^{+} + g_m^{+}\right) + \alpha^{-}\left(g_c^{-} + g_m^{-}\right)} \quad \text{(Equation10)}$$

This final expression depends explicitly only on the geometry of the system. Thus, the positions of kinetochores can be calculated by integrating Equation (10) over time.

Choice of parameters

The value for the stall force of sliding motors f_0 was taken from literature (Valentine et al., 2006) and the value for the sliding motor velocity without a load v_0 was set to reproduce the measured velocity of the bMT and has a similar value to the MT sliding velocity that is driven by Eg5 motor proteins from *Xenopus laevis* (Hentrich and Surrey, 2010). The value for the effective friction coefficient μ_{KC} is estimated as a ratio of the stall force at the kinetochore, which is 3 pN (Akiyoshi et al., 2010) and the polymerization velocity measured here, 0.1 μm/min. Two independent parameters, sliding motor density n_m and passive cross-linker friction multiplied by its density $n_c\mu_c$, were considered as variable parameters and were varied over two orders of magnitude in order to explore the parameter space. Values for geometrical parameters, k-fiber length L_0 and overlap length D_0, were measured here.

Cell culture

Cells were maintained in Dulbecco's Modified Eagle Medium (containing 4.5 g/L d-glucose, stable glutamine, sodium pyruvate; Capricorn Scientific) supplemented with 10% Fetal Bovine Serum (Sigma-Aldrich), 100 IU/mL penicilin and 100 mg/mL streptomycin (Lonza). Cells were grown at 37°C in a Galaxy 170s humidified incubator (Eppendorf) with a 5% CO_2 atmosphere.

RNA interference and transfection

One day before siRNA transfection, 120 000 cells were seeded on 35-mm glass coverslip dishes with 0.17-mm glass thickness (MatTek Corporation). siRNA constructs were diluted in Opti-MEM medium (Gibco) and transfection was performed with Lipofectamine RNAiMAX Reagent (Invitrogen) by following manufacturer's protocol. Constructs and their final concentrations used were: 100 nM Kif18A siRNA (4390825; Ambion), 100 nM Kif4A siRNA (sc-60888; Santa Cruz Biotechnology), 100 nM Kid/Kif22 siRNA (4392420; Ambion), 100 nM CENP-E siRNA (L-003252-000010; Dharmacon), 100 nM MKLP1 siRNA (sc-35936; Santa Cruz Biotechnology), 300 nM PRC1 siRNA (L-019491-00-0010; Dharmacon), 20 nM Haus8 siRNA (L-031247-01-0005; Dharmacon), 100 nM NuMA siRNA (sc-43978; Santa Cruz Biotechnology), and 100 nM Ndc80 siRNA (HA12977117-004; Merck). After 4 h of incubation with transfection mixture, medium was replaced with regular cell culture medium. All experiments on siRNA-treated cells were performed 24 h after transfection, except for Haus8 siRNA-depleted cells, where silencing was done for 48 h. For experiment with spindles devoid of chromosomes, Ndc80 depleted cells were treated with 3 μM ZM447439 (S1103; Selleckchem) and MG-132 inhibitor (474790; Sigma-Aldrich) 30 min before imaging. All treatments include at least three independent experiments. The level of protein depletion per treatment was not correlated with the variability in the flux velocity of k-fibers or bridging fibers (Figure S4I), arguing against the possibility that samples with lower total levels of depletion contained spindles in which targeted proteins were depleted to varying degrees, which would lead to large variability in flux rates.

Speckle microscopy

Cells grown in glass coverslip dishes were stained with 1 nM SiR-tubulin dye (Spirochrome AG). After 15 min of staining, confocal live imaging was performed on a Dragonfly spinning disk confocal microscope system (Andor Technology) using 63x/1.47 HC PL APO glycerol objective (Leica) and Zyla 4.2P scientific complementary metal oxide semiconductor camera (Andor Technology), and Expert Line easy3D STED microscope system (Abberior Instruments) using 60x/1.2 UPLSAPO 60XW water objective (Olympus) and avalanche photodiode detector. Images were acquired using Fusion software and Imspector software. During imaging, cells were maintained at 37°C and 5% CO_2 within heating chamber (Okolab). For live imaging of RPE1 cells expressing CENP-A-GFP and centrin1-GFP, and stained with SiR-tubulin, 488-nm and 640-nm laser lines for Dragonfly microscope system, and 485-nm and 640-nm for Expert Line microscope system were used to excitate GFP, and SiR, respectively. In order to visualize SiR-tubulin speckles, images were acquired with 80% laser power and exposure of 1 s. Image acquisition was done on one focal plane every 5 or 10 s. Note that time-frame within which SiR-tubulin, at 1 nM concentration, can be visualized in patches on the mitotic spindle is between 15 and 75 min after SiR-tubulin staining.

Immunostaining

Cells were fixed in ice-cold methanol for 1 min, except for astrin immunostaining experiment where cells were fixed in 37°C warm 4% paraformaldehyde for 10 min, and permeabilized for 15 min in 0.5% Triton X-100 in PBS. Following permeabilization, cells were blocked with 1% NGS in PBS for 1 h and incubated with primary antibodies at 4°C overnight. Primary antibodies were prepared in 1% NGS in PBS to 1:100 dilution. Following incubation with primary antibodies, cells were incubated with fluorescence-conjugated secondary antibodies at room temperature for 1 h. Secondary antibodies were prepared in 2% NGS in PBS to 1:250 dilution. To visualize DNA, cells were stained with DAPI for 10 min. After each step, cells were washed three times in PBS for 5 min. Primary antibodies used were: rabbit anti-Kif18A (A301-080A; Bethyl Laboratories), mouse anti-Kif4A (sc-365144; Santa Cruz Biotechnology), mouse anti-Kid (sc-390640; Santa Cruz Biotechnology), rabbit anti-CENP-E (C7488; Sigma-Aldrich), rabbit anti-MKLP1 (ab174304; Abcam), mouse anti-PRC1 (sc-376983; Santa Cruz Biotechnology), rabbit anti-Haus8 (PA5-21331; Invitrogen), mouse anti-NuMA (sc-365532; Santa Cruz Biotechnology), and mouse anti-astrin (MABN2487; Sigma-Aldrich). Secondary antibodies used were: donkey anti-mouse IgG-Alexa 594 (Abcam), donkey anti-rabbit IgG-Alexa 594 (Abcam), and donkey anti-rabbit IgG-Alexa 647 (Abcam). Immunostained cells were imaged using Bruker Opterra Multipoint Scanning Confocal Microscope (Bruker Nano Surfaces) with a Nikon CFI Plan Apo VC 100x/1.4 numerical aperture oil objective (Nikon). 405/488/561/640-nm laser lights were used with following emission filters: BL HC 525/30, BL HC 600/37 and BL HC 673/11 (Semrock). Images were captured with an Evolve 512 Delta Electron Multiplying Charge Coupled Device Camera (Photometrics) using a 200 ms exposure time.

Photoactivation assay

For photoactivation experiments, helios one-line 405-nm solid state laser (Obis lasers, Coherent), mounted on Bruker Opterra Multipoint Scanning Confocal Microscope (Buđa et al., 2017), was used to photoactivate MTs in U2OS cells with stable co-expression of photoactivatable-GFP-α-tubulin, CENP-A-GFP and mCherry-α-tubulin. Experiments were performed in *Live/Ablation* mode, at 80% laser power, by using Prairie View software (Prairie Technologies). In order to visualize GFP and mCherry, 488-nm and 561-nm laser lights were used, respectively, together with 250 ms exposure time. K-fibers belonging to same sister kinetochore pairs were sequentially photoactivated when sister kinetochore pairs were displaced from spindle equator, giving rise to shorter and longer sister k-fibers. Images were acquired at one focal plane with a time interval of 2 s.

QUANTIFICATION AND STATISTICAL ANALYSIS

Image analysis

Measurements were performed in Fiji/ImageJ (National Institutes of Health). Quantification and data analysis were performed in R (R Foundation for Statistical Computing) and MATLAB (MathWorks). Figures and schemes were assembled in Adobe Illustrator CC (Adobe Systems). Statistical analysis was performed using Student's t-test, Mann-Whitney test and two-proportions z-test.

Upon inspection of tubulin speckle movement within the spindle, speckles which could be followed for at least 30 s were taken into account. For every tubulin speckle position, corresponding CENP-A and centrin positions, representing the location of sister kinetochores and spindle poles, respectively, were also tracked. Tracking was done by using the *Multi-point* tool. Speckles which started at a proximal kinetochore and were associated with their proximal pole were categorized as a part of k-fiber, whilst speckles which started between sister kinetochores or proximal to the distal pole and passed through sister kinetochores were categorized as a part of bridging fiber. Note that all kinetochore pairs within each spindle were exhaustively inspected for occurrence of k-fiber or bridging fiber speckles, thus the ratio of k-fiber speckles and bridging fiber speckles provides information on the relationship of the number of MTs in these categories (Figure S3C). Speckles that could not be unambiguously categorized as a part of k-fiber or bridging fiber were termed "other" and were the most numerous category. These speckles may or may not belong to MTs that are part of k-fibers or bridging fibers, thus their fraction with respect to k-fiber and bridging fiber speckles in each spindle cannot be used to assess the relative number of different MT subgroups in a straightforward manner. Speckle-pole velocity was calculated by fitting linear regression on distances between the tubulin speckle and the associated spindle pole during first 30 s of its trajectory.

Poleward flux in photoactivation experiments was analyzed by using 5-pixel-thick segmented line to retrieve pole-to-pole GFP and mCherry intensity profiles during 30 s of photoactivated spot movement. Distance between GFP peaks, which correspond to photoactivated tubulin spots, and mCherry peaks, which correspond to spindle poles, was measured over time. By fitting linear regression on distances over 30 s, poleward velocities of photoactivated spots were calculated.

For kinetochore alignment measurements, the *Multipoint* tool was used to track positions of sister kinetochore pairs. The equatorial plane was defined with two points placed between outermost pairs of kinetochores on the opposite sides of the spindle. Kinetochore alignment was calculated as the distance between the midpoint of kinetochore pairs and the equatorial plane.

In cells immunostained for PRC1, a 5-pixel-thick segmented line was used to track the pole-to-pole contour of individual PRC1-labeled overlap regions. The pole-to-pole tracking was performed on single z-planes and the mean value of the cytoplasm was subtracted from the retrieved intensity profiles. The overlap length of individual PRC1-labeled overlap regions (Figures S7F and S7G) was determined as the width of the peak of the signal intensity in the central part of the contour in SciDavis (Free Software Foundation Inc.). The width of the peak was measured at the base of the PRC1 intensity peak where the PRC1 signal is roughly equal to the mean value of the PRC1 signal along the contour on either side of the peak. Similarly, by using *Line* tool a 100-pixel-thick line was used to retrieve the pole-to-pole profiles of PRC1 intensity within whole spindles. This was done on sum intensity projection of

five z-planes. The overlap length of PRC1-labeled overlap regions within whole spindles (Figures 5A and S7E) was determined as the width of the peak at the half-height of each peak.

To determine the percentage of protein depletion, we measured mean spindle intensity by encompassing the area of the spindle with the *Polygon selection* tool. Mean background intensity in the cytoplasm, measured using a 1 × 1 μm rectangle, was subtracted from the mean spindle intensity.

Cell Reports, Volume *40*

Supplemental information

Length-dependent poleward flux of sister kinetochore fibers promotes chromosome alignment

Patrik Risteski, Domagoj Božan, Mihaela Jagrić, Agneza Bosilj, Nenad Pavin, and Iva M. Tolić

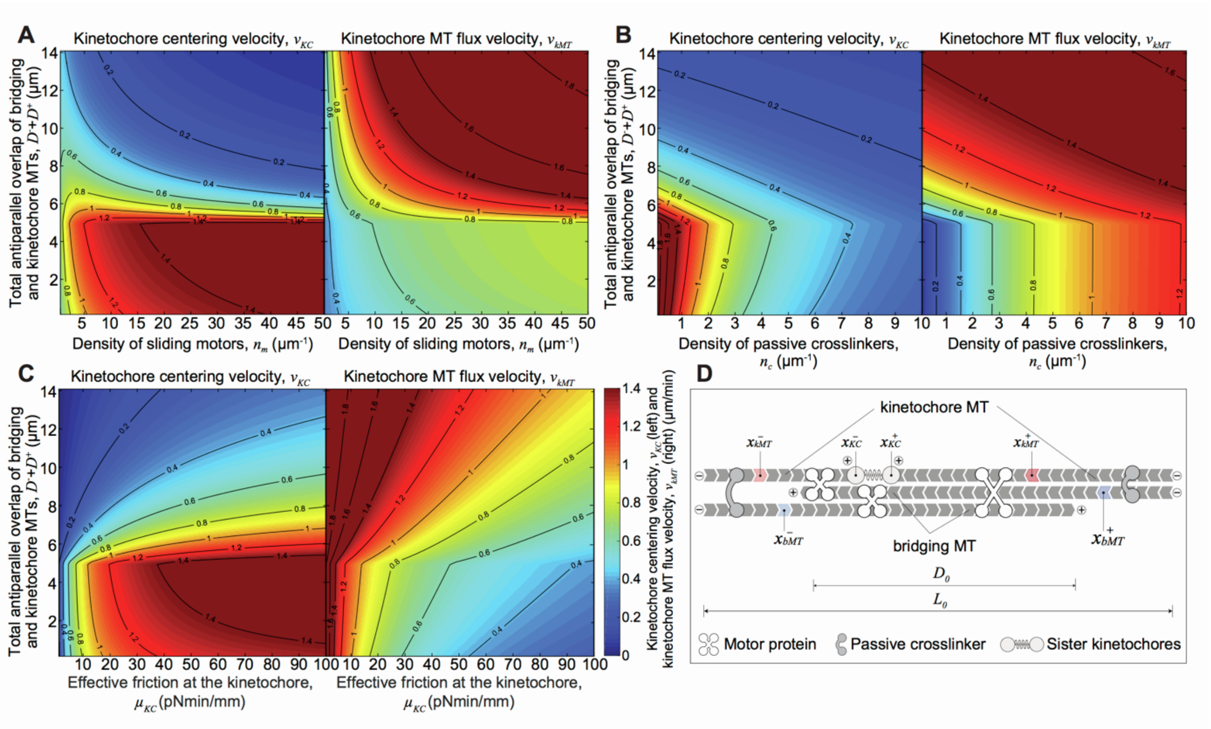

Figure S1. Centering velocity and k-fiber flux for different parameters, Related to Figure 1. Kinetochore centering velocity (left) and kMT flux velocity (right) for different values of motor density (**A**), crosslinker density (**B**), and effective friction at the kinetochore (**C**), and different values of the length of total antiparallel overlap of bMTs and kMTs. In all cases, faster centering velocities correlate with slower k-fiber flux velocities and vice versa and thus we comment transitions for centering velocities only. (**A**) Data shows two distinct regimes of centering velocity: fast centering velocities for shorter overlaps (red region) which sharply decreases when the overlap length exceeds 4 μm (blue region). This abrupt change occurs when the shorter k-fiber loses connection with antiparallel bMT and thus there is no motor force that opposes centering movement. Transition between these two regimes is less abrupt for lower values of motor densities ($n_{\mathrm{m}} < 10\ \mu\mathrm{m}^{-1}$). (**B**) Similar to panel **A**, fast centering velocities are obtained for shorter overlaps (red region). However, the centering velocity decreases with the increase in crosslinker density, irrespective of the overlap length (blue region). (**C**) Fast centering velocities are obtained for shorter overlaps (red region) and the centering velocity increases with the increase in effective friction. For low values of effective friction, both the centering velocity and the k-fiber flux velocity retain similar values regardless of the overlap length. Bar on the right denotes the relationship between color and velocity values for panels **A**-**C**. (**D**) Top: Scheme of the model. KMTs extend from the edges toward elastically connected kinetochores and bMTs extend from the edges towards each other. Motor proteins connect antiparallel MTs, while passive crosslinkers connect parallel MTs. Total lengths of antiparallel

and parallel MT overlaps are denoted as D_0 and L_0, respectively. Positions of sister kinetochores are marked on the x-axis. Positions of k-fibers (red) and bridging fibers (blue) are taken as arbitrary positions along their lattice and are also marked on the x-axis. Superscripts + and – denote the right and left sides, respectively. Bottom: Legend describing symbols for different elements of the spindle in the scheme. Parameters for all panels are given in **Figure 1B** and the initial position of kinetochores is $x_{\mathrm{KC}} = -2$ µm, if not stated otherwise.

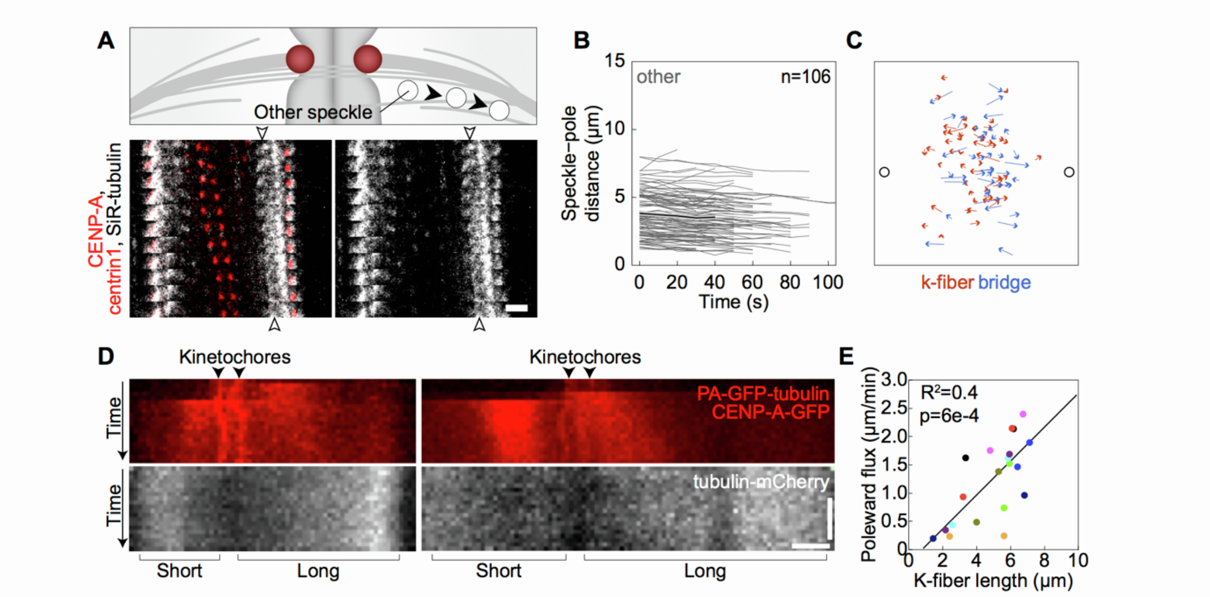

Figure S2. K-fiber poleward flux correlates with k-fiber length, Related to Figure 2. (**A**) Scheme of "other" speckles for which it could not be determined the type of MT they belong to (top). Montage over time demonstrating the movement of this group of speckles. Merge (left); tubulin channel only (right). Arrowheads mark starting and ending positions of the tracked speckle. Scale bar: 2 µm. (**B**) Distance of "other" speckles from the pole over time in untreated cells. Gray lines show individual speckles. Black line; mean. Gray area; SEM (**C**) Examples of trajectories of speckles belonging to k-fibers (red) and bridging fibers (blue) within 30 s of their movement. Arrows are pointing towards corresponding direction. Black circles; spindle poles. (**D**) Kymographs retrieved by pole-to-pole segmented lines in U2OS cells stably co-expressing PA-GFP-α-tubulin (red), CENP-A-GFP (red) and mCherry-α-tubulin (gray) during poleward motion of the photoactivated spots on shorter and longer sister k-fibers. Horizontal scale bar, 2 µm; vertical scale bar, 20 s. (**E**) Graph shows poleward flux of photoactivated spots in U2OS cells with respect to their corresponding k-fiber length, color-coded for each sister k-fiber pair as in **Figure 2I**.

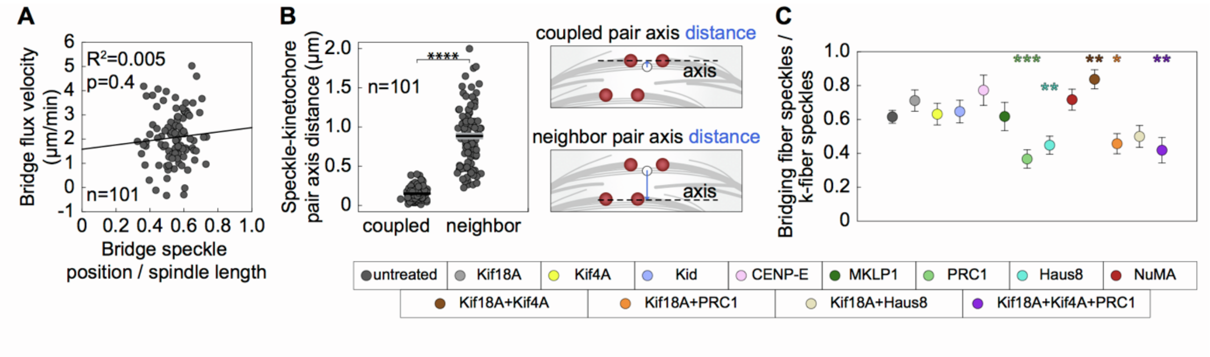

Figure S3. Characterization of the bridging fiber poleward flux, Related to Figure 3. (**A**) Poleward velocity of bridging fiber speckles depending on their relative starting speckle-pole distance. Starting position of all bridging fiber speckles was close to the position of associated kinetochore pair. (**B**) Distances between tracked bridging fiber speckle and kinetochore pair they were associated to and between tracked bridging fiber speckle and their closest neighboring kinetochore pair. Schematics represent how these distances were measured. Black lines, mean; gray areas, SEM. (**C**) Ratio of tracked speckles within bridging fibers and k-fibers (top) color-coded for corresponding treatments as in legend (bottom). In **C**, each treatment is compared with untreated cells. Treatments include at least three independent experiments. Statistical analysis conducted using t-test (**B**) and two-proportions z-test (**C**); p values as in **Figure 3**.

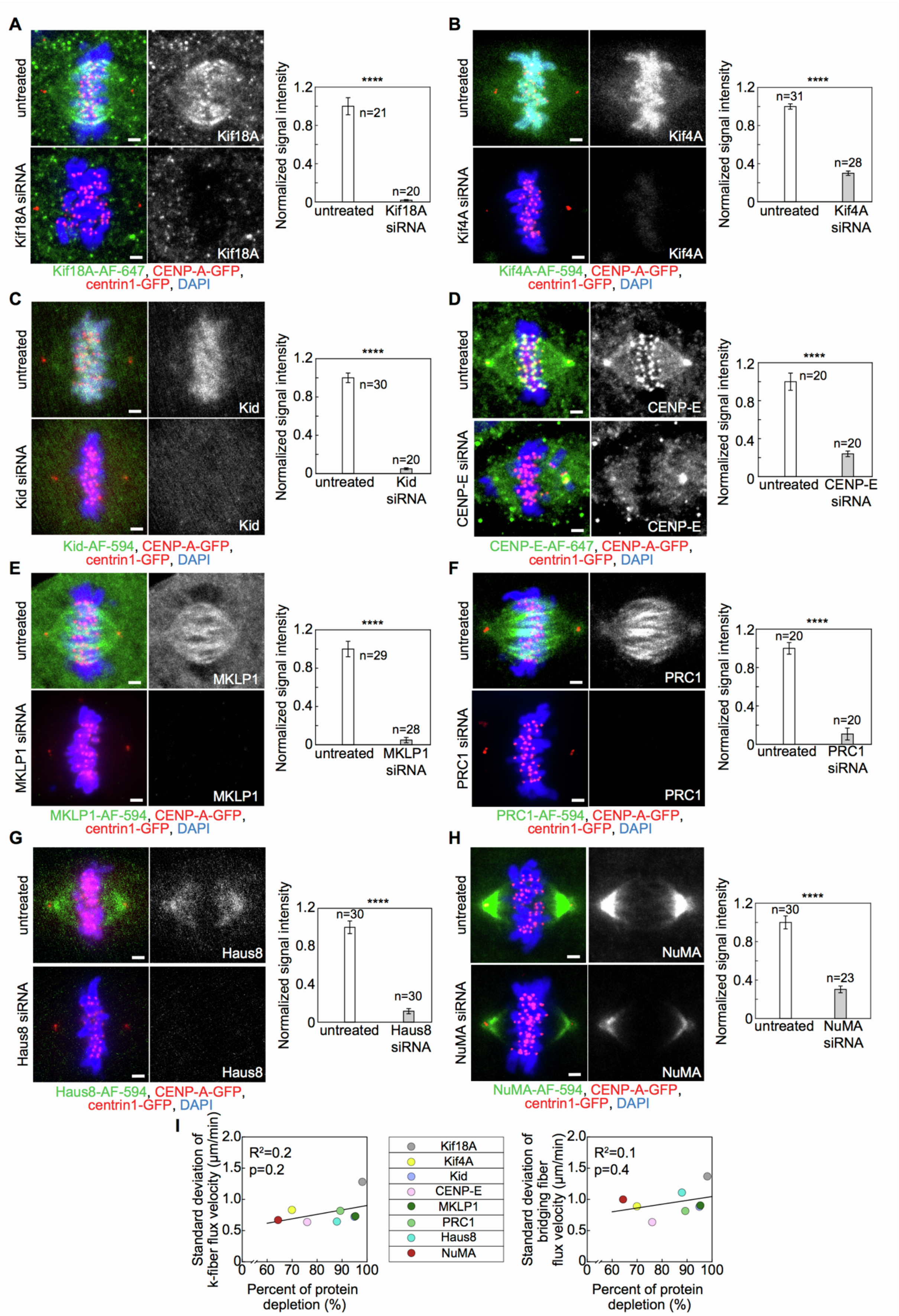

A
untreated
Kif18A siRNA
Kif18A
Kif18A-AF-647, CENP-A-GFP, centrin1-GFP, DAPI
Normalized signal intensity
****
n=21
n=20
untreated
Kif18A siRNA
B
untreated
Kif4A siRNA
Kif4A
Kif4A-AF-594, CENP-A-GFP, centrin1-GFP, DAPI
n=31
n=28
Kif4A siRNA
C
Kid siRNA
Kid
Kid-AF-594, CENP-A-GFP, centrin1-GFP, DAPI
n=30
n=20
Kid siRNA
D
CENP-E siRNA
CENP-E
CENP-E-AF-647, CENP-A-GFP, centrin1-GFP, DAPI
n=20
n=20
CENP-E siRNA
E
MKLP1 siRNA
MKLP1
MKLP1-AF-594, CENP-A-GFP, centrin1-GFP, DAPI
n=29
n=28
MKLP1 siRNA
F
PRC1 siRNA
PRC1
PRC1-AF-594, CENP-A-GFP, centrin1-GFP, DAPI
n=20
n=20
PRC1 siRNA
G
Haus8 siRNA
Haus8
Haus8-AF-594, CENP-A-GFP, centrin1-GFP, DAPI
n=30
n=30
Haus8 siRNA
H
NuMA siRNA
NuMA
NuMA-AF-594, CENP-A-GFP, centrin1-GFP, DAPI
n=30
n=23
NuMA siRNA
0 0.4 0.8 1.2
I
Standard deviation of k-fiber flux velocity (µm/min)
R²=0.2
p=0.2
Percent of protein depletion (%)
0 60 70 80 90 100
0 0.5 1.0 1.5 2.0
Kif18A
Kif4A
Kid
CENP-E
MKLP1
PRC1
Haus8
NuMA
Standard deviation of bridging fiber flux velocity (µm/min)
R²=0.1
p=0.4

Figure S4. Depletion efficiencies for siRNA treatments, Related to Figure 3. (**A**)-(**H**) Fixed spindles in RPE1 cell line stably expressing CENP-A-GFP and centrin1-GFP (red) in cells immunostained for (**A**) Kif18A (AF-647, green), (**B**) Kif4A (AF-594, green), (**C**) Kid (AF-594, green), (**D**) CENP-E (AF-647, green), (**E**) MKLP1 (AF-594, green), (**F**) PRC1 (AF-594, green), (**G**) Haus8 (AF-594, green) and (**H**) NuMA (AF-594, green) in untreated (upper rows) and corresponding siRNA-treated cells (bottom rows), with DNA stained with DAPI (blue). Left: merge; right: protein of interest (gray). Graphs showing intensities of indicated proteins in untreated (white bars) and siRNA treated (gray bars) cells are given on the right. All values are normalized to the mean intensity value of untreated cells for each protein. All treatments include at least two independent experiments. n; number of cells. Scale bars; 2 μm. All images are maximum intensity projections of five z-planes smoothed with 0.5-pixel-sigma Gaussian blur. (**I**) Standard deviation of k-fiber (left) and bridging fiber (right) flux velocity with respect to the level of protein depletion in corresponding siRNA-treatments (legend). Statistical analysis conducted using t-test; p values as in **Figure 3**.

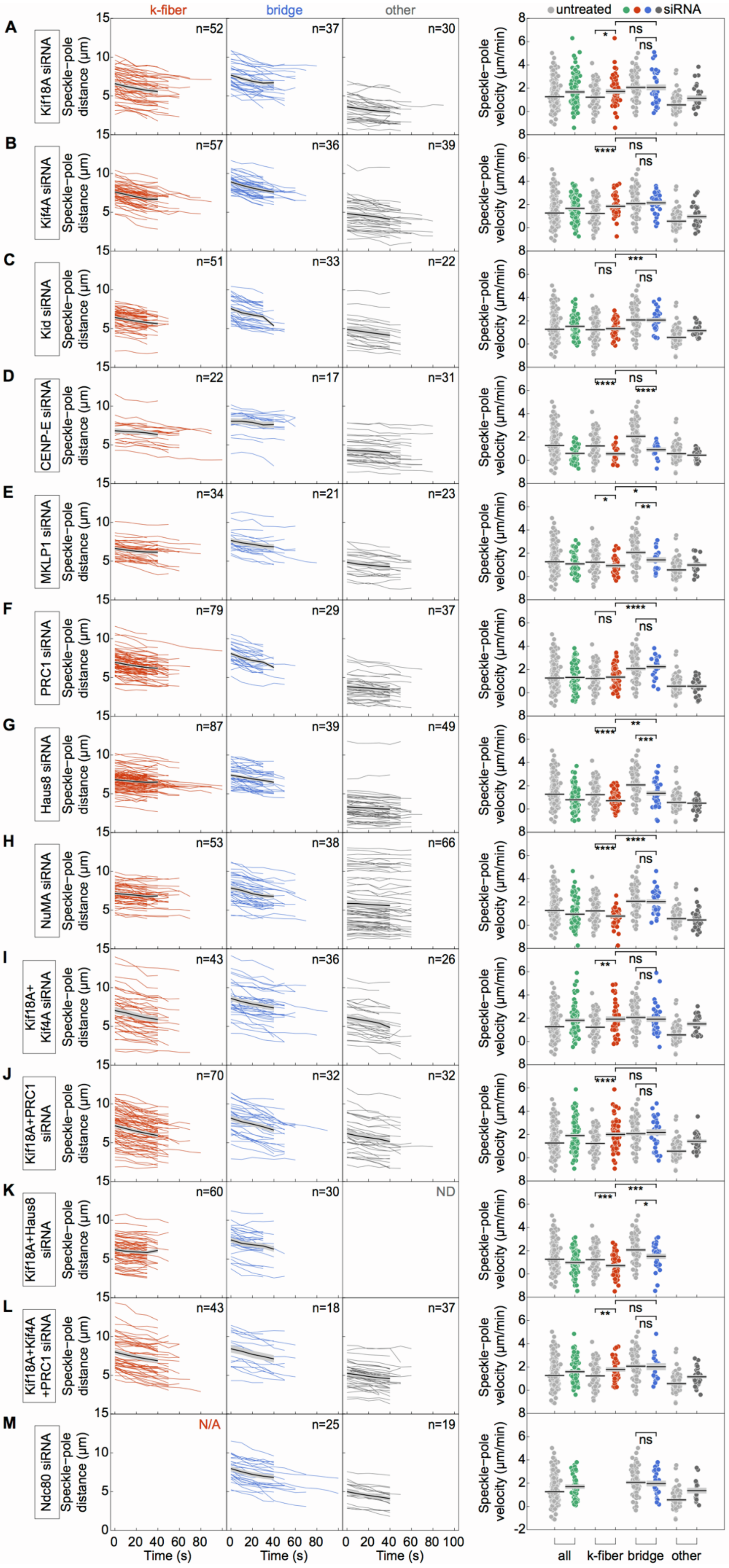

k-fiber
bridge
other
untreated
siRNA
A
Kif18A siRNA
B
Kif4A siRNA
C
Kid siRNA
D
CENP-E siRNA
E
MKLP1 siRNA
F
PRC1 siRNA
G
Haus8 siRNA
H
NuMA siRNA
I
Kif18A+ Kif4A siRNA
J
Kif18A+PRC1 siRNA
K
Kif18A+Haus8 siRNA
L
Kif18A+Kif4A +PRC1 siRNA
M
Ndc80 siRNA
Speckle−pole distance (μm)
Speckle−pole velocity (μm/min)
Time (s)
N/A
ND
all
k-fiber
bridge
other

Figure S5. Comparison of microtubule poleward flux rates per treatment, Related to Figure 3. Poleward flux after depletion of (**A**) Kif18A, (**B**) Kif4A, (**C**) Kid, (**D**) CENP-E (**E**) MKLP1, (**F**) PRC1, (**G**) Haus8, (**H**) NuMA, (**I**) Kif18A+Kif4A, (**J**) Kif18A+PRC1, (**K**) Kif18A+Haus8, (**L**) Kif18A+Kif4A+PRC1 and (**M**) Ndc80. Graphs from left to right show: speckles on kMTs, speckles on bMTs, and other speckles. Colored lines show individual speckles. Black lines; mean. Gray areas; SEM. Poleward velocity of the speckles is shown at the right. Black lines; mean. Gray areas; SEM. One outlier in untreated cells is not shown. All treatments include at least three independent experiments. n; number of measurements. Statistical analysis conducted using t-test; p values as in **Figure 3**.

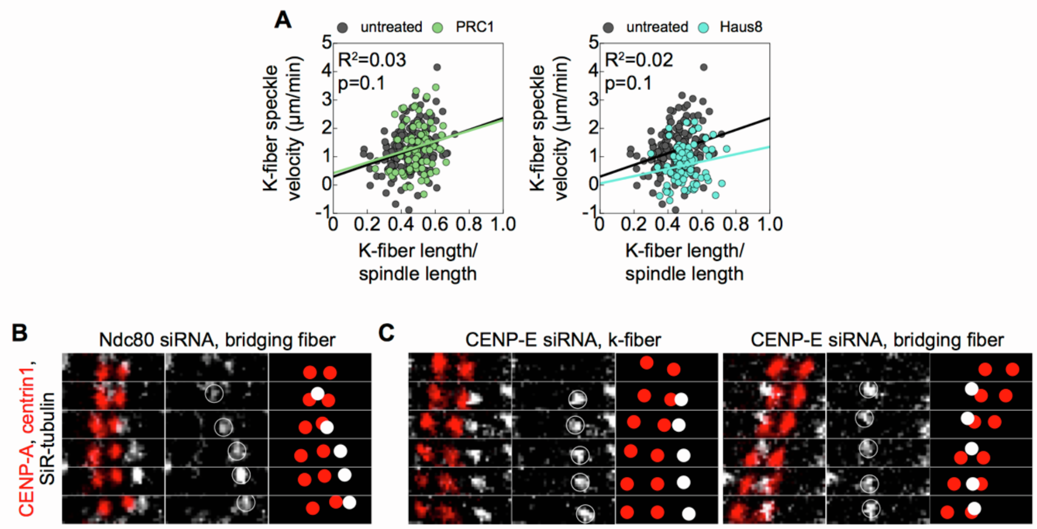

Figure S6. Effect of the bridging microtubule sliding on k-fiber poleward flux, Related to Figure 3. (**A**) Poleward velocity of k-fiber speckles depending on their relative starting speckle-pole distance in PRC1 (left) and Haus8 (right) siRNA-treated cells. (**B**) Montage over time demonstrating the movement of a speckle belonging to the bridging fiber in Ndc80 siRNA treatment. (**C**) Montage over time demonstrating the movement of a speckle belonging to the k-fiber (left) and bridging fiber (right) in CENP-E siRNA treatment. Legend as in **Figure 2B**.

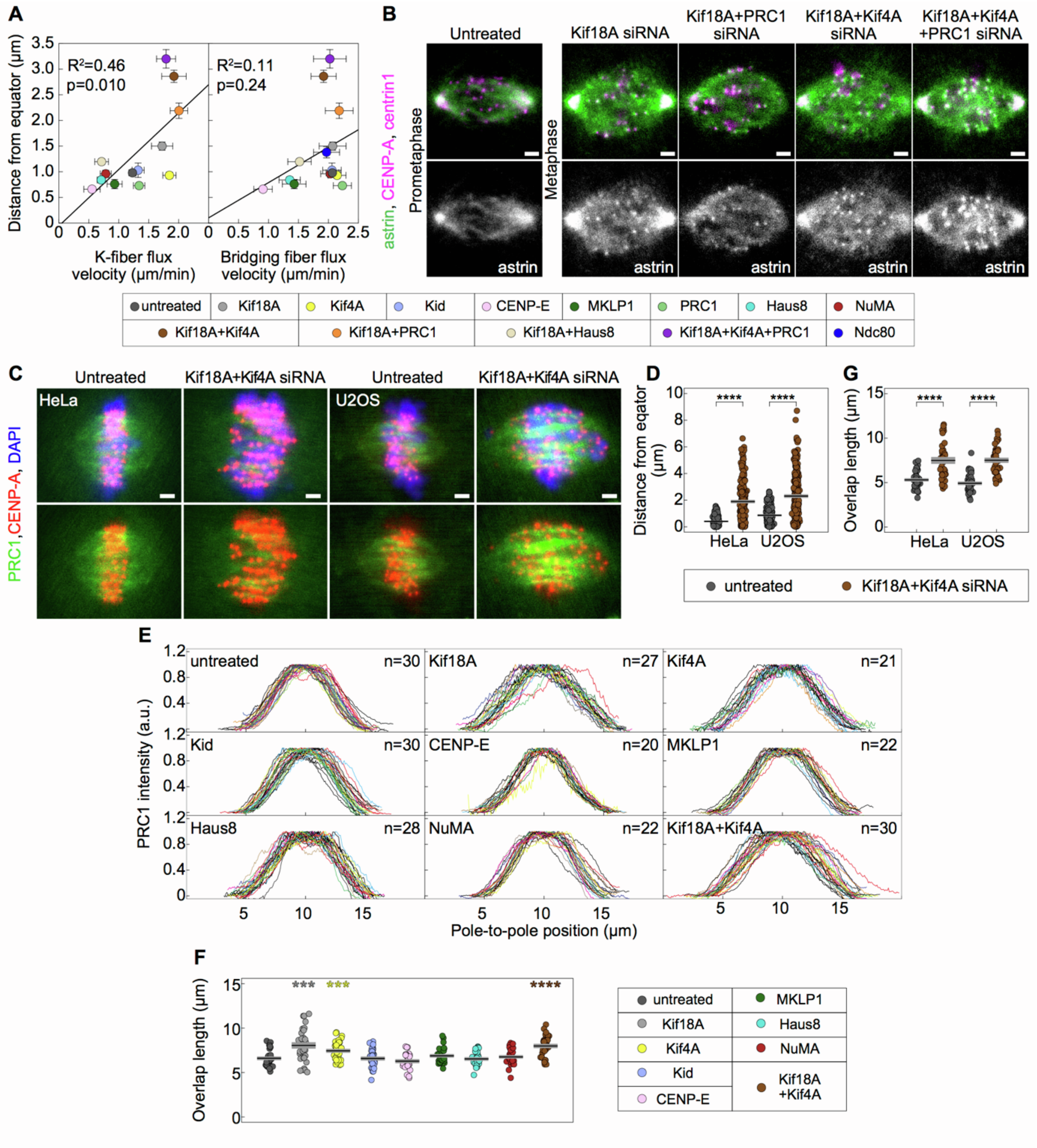

Figure S7. Longer antiparallel overlaps lead to increased k-fiber flux velocity and kinetochore misalignment, Related to Figures 4 and 5. (**A**) Kinetochore distance from equator versus k-fiber (left) and bridging fiber (right) flux velocity in untreated and siRNA-treated cells. (**B**) Fixed spindles in RPE1 cells stably expressing CENP-A-GFP and centrin1-GFP (magenta), immunostained for astrin (AF-594, green) in untreated and treated with Kif18A, Kif18A and PRC1, Kif18A and Kif4A, and Kif18A, Kif4A and PRC1 siRNA (left to right). Top: merge; bottom: astrin (gray). Images are sum intensity projections of five z-planes. (**C**) Fixed spindles in HeLa and U2OS cells stably expressing CENP-A-GFP (red) in untreated

(left) and Kif18A and Kif4A siRNA treated cells (right), immunostained for PRC1 (AF-594, green) and stained with DAPI (blue). Top: merge; bottom: only CENP-A and PRC1. Images are maximum intensity projections of five z-planes. (**D**) Kinetochore distance from equator in untreated and Kif18A and Kif4A siRNA treated HeLa (n = 172 and n = 235 kinetochore pairs) and U2OS (n = 216 and n = 281 kinetochore pairs) cells. Black lines, mean. Gray areas; SEM. (**E**) Normalized pole-to-pole PRC1 intensity profiles of complete spindles for given treatments. Lines correspond to individual spindles. (**F**) Length of individual PRC1-labeled overlaps. siRNA treatments are color-coded according to the legend. Black lines, mean. Gray areas; SEM. (**G**) Length of individual PRC1-labeled overlaps in untreated and Kif18A and Kif4A siRNA treated HeLa (n = 46 and n = 49 PRC1 bundles) and U2OS (n = 47 and n = 41 PRC1 bundles) cells. Black lines, mean. Gray areas; SEM. In **F**, each treatment is compared with untreated cells. Treatments in **A**, **D**, **F**, **G** are color-coded according to the legend. Statistical analysis conducted using t-test in **F**, **G**, and the Mann-Whitney test in **D**; p values as in **Figure 3**.
